# p300 or CBP is required for insulin-stimulated glucose uptake in skeletal muscle and adipocytes

**DOI:** 10.1172/jci.insight.141344

**Published:** 2022-01-11

**Authors:** Vitor F. Martins, Samuel A. LaBarge, Alexandra Stanley, Kristoffer Svensson, Chao-Wei Hung, Omer Keinan, Theodore P. Ciaraldi, Dion Banoian, Ji E. Park, Christina Ha, Byron Hetrick, Gretchen A. Meyer, Andrew Philp, Larry L. David, Robert R. Henry, Joseph E. Aslan, Alan R. Saltiel, Carrie. E. McCurdy, Simon Schenk

**Affiliations:** 1 Department of Orthopedic Surgery and; 2 Biomedical Sciences Graduate Program, University of California, San Diego, La Jolla, California, USA.; 3 Department of Medicine, University of California San Diego School of Medicine, La Jolla, California, USA.; 4 VA San Diego Healthcare System, San Diego, California, USA.; 5 Department of Human Physiology, University of Oregon, Eugene, Oregon, USA.; 6 Program in Physical Therapy, Washington University in St. Louis, St. Louis, Missouri, USA.; 7 Garvan Institute of Medical Research, Darlinghurst, New South Wales, Australia.; 8 St Vincent’s Clinical School, UNSW Medicine, UNSW Sydney, Sydney, New South Wales, Australia.; 9 Department of Biochemistry and Molecular Biology,; 10 Knight Cardiovascular Institute, and; 11 Department of Biomedical Engineering, School of Medicine, Oregon Health & Science University, Portland, Oregon, USA.

**Keywords:** Endocrinology, Muscle Biology, Glucose metabolism, Insulin signaling, Skeletal muscle

## Abstract

While current thinking posits that insulin signaling to glucose transporter 4 (GLUT4) exocytic translocation and glucose uptake in skeletal muscle and adipocytes is controlled by phosphorylation-based signaling, many proteins in this pathway are acetylated on lysine residues. However, the importance of acetylation and lysine acetyltransferases to insulin-stimulated glucose uptake is incompletely defined. Here, we demonstrate that combined loss of the acetyltransferases E1A binding protein p300 (p300) and cAMP response element binding protein binding protein (CBP) in mouse skeletal muscle caused a complete loss of insulin-stimulated glucose uptake. Similarly, brief (i.e., 1 hour) pharmacological inhibition of p300/CBP acetyltransferase activity recapitulated this phenotype in human and rodent myotubes, 3T3-L1 adipocytes, and mouse muscle. Mechanistically, these effects were due to p300/CBP-mediated regulation of GLUT4 exocytic translocation and occurred downstream of Akt signaling. Taken together, we highlight a fundamental role for acetylation and p300/CBP in the direct regulation of insulin-stimulated glucose transport in skeletal muscle and adipocytes.

## Introduction

Current thinking posits that phosphorylation-based signaling regulates insulin stimulation to glucose transporter 4 (GLUT4) exocytic translocation and glucose uptake, in both skeletal muscle and adipose tissue (1, 2). However, recent acetylomics studies have revealed that many proteins key to both insulin signaling and GLUT4 exocytic translocation can be reversibly acetylated on lysine residues (3–5). Classically, lysine acetylation is known to regulate biological processes through transcription via acetylation of histones and transcription factors (6). However, like phosphorylation, acetylation can regulate the function of proteins in essentially all cellular compartments via direct structural conformational change, including proteins important to metabolism (6). Reversible lysine acetylation requires the enzymatic addition or removal of acetyl groups on lysine residues by acetyltransferases or deacetylases, respectively (6). While the deacetylases have been studied extensively for both their transcriptional (7–9) and nontranscriptional (10, 11) control of insulin action, the contribution of the acetyltransferases remains poorly understood.

The acetyltransferases E1A binding protein p300 (p300) and cAMP response element binding protein (CREB) binding protein (CBP) are functional homologs that have a strong nuclear and cytosolic presence (12–14). Their cytosolic presence allows them to acetylate and directly regulate the function of a variety of cytosolic proteins, including those that are important to insulin action (15–18). For example, within the proximal insulin signaling pathway, p300/CBP can acetylate Rictor, increasing its activity in HeLa cells (17). Similarly, p300/CBP can acetylate both insulin receptor substrate 1 in Hep1-6 cells (16), and Akt (15) in both HeLa and HEK293T cells, with acetylation of these proteins decreasing their activity. Furthermore, a recent study identifying the p300/CBP-regulated acetylome in mouse embryonic fibroblasts identified a number of proteins known to contribute to GLUT4 exocytic translocation, including myosin 1C and exocyst complex 2 and 3 (18). Importantly, however, none of these studies investigated the relevance of p300/CBP acetyltransferase activity to insulin action. To this point, p300/CBP are phosphorylated in response to insulin stimulation in HeLa cells, HEK293T cells, and mouse liver (19–21). Moreover, p300/CBP are phosphorylated by Akt, which increases their acetyltransferase activity in a variety of cell types (13, 22, 23). Considered together, these findings suggest that p300/CBP may be an important regulator of insulin signaling to glucose uptake.

The primary goal of this study was to investigate the combined contribution of p300 and CBP to insulin-mediated glucose uptake. We demonstrate that blocking p300/CBP activity, either by gene knockout in mice or by brief pharmacological inhibition, abolished insulin-stimulated acetylation in rodent myoblasts and insulin-stimulated glucose uptake in human and rodent myotubes, adipocytes, and mature mouse muscle. Importantly, these effects on insulin-stimulated glucose uptake occurred in conjunction with inhibition of insulin-stimulated GLUT4 exocytic translocation. Taken together, these data demonstrate that p300/CBP and acetylation are potentially novel and integral regulators of insulin-stimulated glucose uptake and that this regulation occurs independent of their well-known role as transcriptional regulators.

## Results

### Severe skeletal muscle insulin resistance develops rapidly in mice with skeletal muscle–specific knockout of both p300 and CBP.

We have previously demonstrated that individual, muscle-specific, and inducible knockout of p300 or CBP in mice does not impact glucose tolerance or skeletal muscle insulin signaling and sensitivity (24). We attributed this lack of effect to the well-documented compensatory actions of p300 and CBP that occur when only p300 or CBP is present (25, 26). Thus, we interbred these individual knockout models to generate a p300 and CBP skeletal muscle–specific, tamoxifen-inducible, double-knockout mouse (referred to as PCKO), which we previously described (27). Because PCKO mice die 6–7 days after initiating tamoxifen treatment (27), in this study all experiments in PCKO mice and wild-type (WT) littermates were conducted at day 1 (D1), 3 (D3), or 5 (D5) after starting tamoxifen dosing.

**Figure 1:**
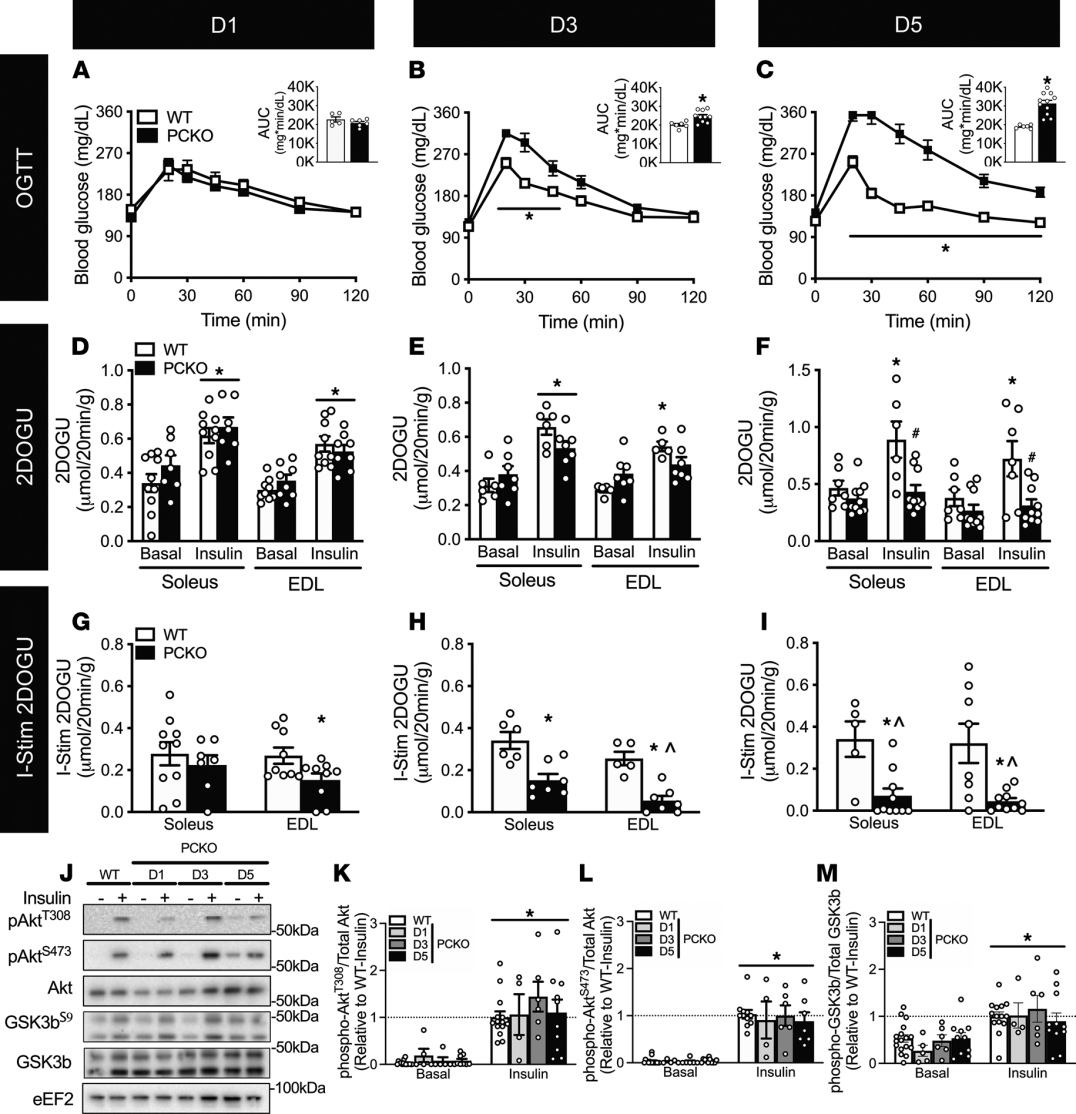
p300 and CBP are required for skeletal muscle insulin-stimulated glucose uptake. Male PCKO mice were assessed at 1 (D1), 3 (D3), or 5 (D5) days after initiating tamoxifen. (**A**–**C**) Blood glucose concentrations and area under the curve (AUC; inset) for male PCKO mice at D1, D3, and D5 during an oral glucose tolerance test (OGTT; 2 g/kg); D1: WT/PCKO *n* = 5/9, D3: WT/PCKO *n* = 6/10, D5: WT/PCKO *n* = 6/11. *, *P* < 0.05 2-way ANOVA repeated measures, PCKO versus WT within a time point for OGTT, and *, *P* < 0.05 *t* test for AUC. (**D**–**F**) Basal 2-deoxy-glucose uptake (2DOGU), insulin (0.36 nmol/L) 2DOGU, and (**G**–**I**) insulin-stimulated 2DOGU (I-Stim.; calculated as insulin 2DOGU – basal 2DOGU) in isolated soleus and extensor digitorum longus (EDL) muscles from male WT and PCKO mice at D1, D3, and D5; D1: WT/PCKO *n* = 11/10, D3: WT/PCKO *n* = 6/7, D5: WT/PCKO *n* = 7/10. *, *P* < 0.05 2-way ANOVA with Sidak’s multiple comparison versus basal within genotype (**D**–**F**) and ^#^, *P* < 0.05 versus WT within basal or insulin (**G**–**I**). *, *P* < 0.05 *t* test for I-Stim. ^, *P* > 0.05 1-sample *t* test versus “0”. (**J**) Representative images for phospho-Akt^T308^ (pAkt^T308^), phospho-Akt^S473^ (pAkt^S473^), total Akt, phospho-GSK3b^S9^ (pGSK3b^S9^), and total GSK3b in basal and insulin-stimulated (- and +, respectively) EDL muscles from WT and PCKO mice at D1, D3, and D5. Quantification of (**K**) pAkt^T308^, (**L**) pAkt^S473^, and (**M**) pGSK3b^S9^ compared with total respective protein abundance in the EDL muscle; WT/D1/D3/D5 *n* = 15/5/6/11. Values are presented relative to WT-insulin. *, *P* < 0.05 2-way ANOVA, main effect of insulin. Data reported as mean ± SEM. For Western blots, there were no significant differences between WT mice at the different time points (D1, D3, and D5); therefore WT data were collapsed.

Initially, we temporally studied body weight and glucose tolerance in the same cohort of male PCKO and WT mice. Body weights were comparable between WT and PCKO mice on D1 and D3; however, by D5, PCKO mice were significantly lighter than WT mice (Supplemental Figure 1A; supplemental material available online with this article; https://doi.org/10.1172/jci.insight.141344DS1). While glucose tolerance was comparable between PCKO and WT mice at D1 (Figure 1A), by D3 and especially D5, PCKO mice were severely glucose intolerant (Figure 1, B and C); by D5 the AUC during the oral glucose tolerance test (OGTT) was approximately 50% higher in PCKO versus WT mice (Figure 1, A–C, inset). There was no difference in fasting insulin concentration in PCKO versus WT mice (Supplemental Figure 1B). Since our mouse line is a skeletal muscle–specific knockout, we next studied whether skeletal muscle insulin action similarly deteriorated over time. For this, we studied basal and insulin-stimulated 2-deoxyglucose (2DOG) uptake using a physiological insulin concentration (0.36 nM) in the soleus (predominantly slow twitch) and extensor digitorum longus (EDL; predominantly fast twitch) muscles; studying these 2 muscles is advantageous as it also allows evaluation of potential fiber type–specific differences. 2DOG uptake in the presence of insulin, and “insulin-stimulated” 2DOG uptake (i.e., insulin 2DOG uptake minus basal 2DOG uptake) were comparable in the soleus of PCKO and WT mice at D1 (Figure 1, D and G). However, insulin-stimulated 2DOG uptake in the soleus was approximately 50% lower in PCKO mice compared with WT mice by D3 (Figure 1, E and H), and by D5 it was inhibited such that it was not different from basal 2DOG uptake (Figure 1, F and I). These effects were even more rapid in the EDL with insulin-stimulated 2DOG uptake being approximately 50% lower on D1 and not significantly different from basal by D3 and D5 (Figure 1, G–I). In both soleus and EDL, these effects on insulin-stimulated 2DOG uptake in PCKO mice were driven by an inability of muscle to respond to insulin as basal 2DOG uptake was not different from WT mice at any time point. Interestingly, these severe impairments in insulin-stimulated glucose uptake were not associated with a change in proximal insulin signaling, as insulin stimulation equally increased phosphorylation of Akt^T308^, Akt^S473^, and GSK3b^S9^ (glycogen synthase kinase 3 β) regardless of genotype or day (Figure 1, J–M).

### A single allele of p300 or CBP rescues glucose intolerance and the loss of skeletal muscle insulin-stimulated glucose uptake seen in PCKO mice.

**Figure 2:**
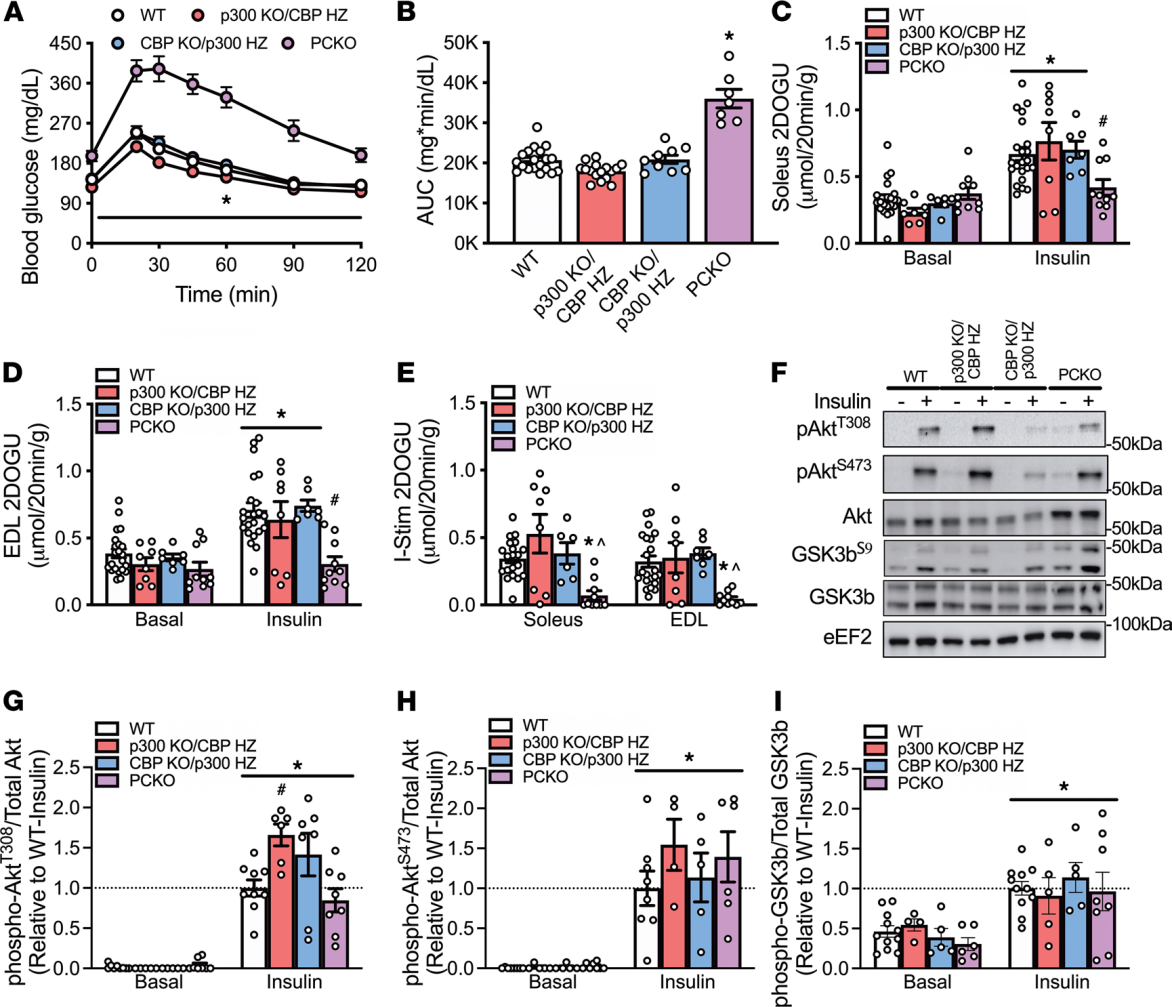
Mice with a single allele of either p300 or CBP have normal glucose tolerance and skeletal muscle insulin action. Male WT, p300 KO/CBP HZ, CBP KO/p300 HZ, and PCKO mice were assessed at 5 days after initiating tamoxifen. (**A**) Blood glucose concentrations and (**B**) AUC for male WT, p300 KO/CBP HZ, CBP KO/p300 HZ, and PCKO mice during an OGTT (2 g/kg); for WT, p300 KO/CBP HZ, CBP KO/p300 HZ, PCKO *n* = 18/16/9/7. *, *P* < 0.05 2-way ANOVA with repeated measures, PCKO versus WT within a time point for OGTT (**A**); *, *P* < 0.05 1-way ANOVA, versus WT for AUC (**B**). (**C** and **D**) Basal 2DOGU, insulin (0.36 nmol/L) 2DOGU, and (**E**) insulin-stimulated 2DOGU (calculated as insulin 2DOGU – basal 2DOGU) in isolated soleus and EDL muscles from male WT, p300 KO/CBP HZ, CBP KO/p300 HZ, and PCKO mice; WT, p300 KO/CBP HZ, CBP KO/p300 HZ, PCKO *n* = 20/8/7/10. *, *P* < 0.05 2-way ANOVA with Sidak’s multiple comparison versus basal within genotype and ^#^, *P* < 0.05 versus WT within basal or insulin (**C** and **D**). For I-Stim (**E**), *, *P* < 0.05 1-way ANOVA with Tukey’s multiple comparison versus WT. ^, *P* > 0.05 1-sample *t* test versus “0.” (**F**) Representative images for pAkt^T308^, pAkt^S473^, total Akt, pGSK3b^S9^, and total GSK3b in basal and insulin-stimulated (- and +, respectively) EDL muscles from WT, p300 KO/CBP HZ, CBP KO/p300 HZ, and PCKO mice. Quantification of (**G**) pAkt^T308^, (**H**) pAkt^S473^, and (**I**) pGSK3b^S9^ compared with total respective protein abundance in the EDL muscle; WT, p300 KO/CBP HZ, CBP KO/p300 HZ, PCKO *n* = 8/5/5/6. Values are presented relative to WT-insulin. *, *P* < 0.05 2-way ANOVA, main effect of insulin. ^#^, *P* < 0.05 2-way ANOVA, multiple comparison versus WT-insulin. Data reported as mean ± SEM. For all data, there were no significant differences between WT mice for the respective lines (p300 KO/CBP HZ, CBP KO/p300 HZ, and PCKO); therefore, WT data were collapsed.

To determine dosage effects of p300 and CBP on insulin action, using a single-allele rescue approach, we studied male and female mice with just 1 allele of p300 or CBP and knockout of the other protein (i.e., just 1 of 4 possible alleles and referred to as CBP KO/p300 HZ and p300 KO/CBP HZ, respectively) in skeletal muscle and their respective WT littermates. Concurrently, we studied a separate cohort of PCKO mice with measures being made on D5 after initiating tamoxifen dosing; D5 was chosen as there was a robust abrogation of insulin-stimulated glucose uptake by skeletal muscle in PCKO mice at this time point. The CBP KO/p300 HZ and p300 KO/CBP HZ lines have been described previously (27). Notably, each mouse line (p300 KO/CBP HZ, CBP KO/p300 HZ, and PCKO) had its own WT littermate controls; however, because there were no significant differences between these WT lines in glucose tolerance or insulin-stimulated glucose uptake (Supplemental Figure 2), we collapsed them into 1 group. As expected, PCKO mice had lower body weight and were severely glucose intolerant, and insulin-stimulated glucose uptake in soleus and EDL muscles was not different from basal (male: Figure 2, A–E, and Supplemental Figure 1C; female: Supplemental Figure 1D and Supplemental Figure 3). Remarkably, however, the single-allele rescue of p300 or CBP in CBP KO/p300 HZ and p300 KO/CBP HZ, respectively, was sufficient to normalize body weight, glucose tolerance, and skeletal muscle insulin action to those seen in WT mice, regardless of muscle type or sex (male: Figure 2, A–E, and Supplemental Figure 1C; female: Supplemental Figure 1D and Supplemental Figure 3). Complementing the 2DOG uptake data, insulin significantly increased phosphorylation of Akt^T308^, Akt^S473^, and GSK3b^S9^ in p300 KO/CBP HZ, CBP KO/p300 HZ, and PCKO mice to at least the level of WT littermates (Figure 2, F–I).

### PCKO mouse muscles have substantial changes in mRNA expression of genes involved in insulin signaling and GLUT4 trafficking.

**Figure 3:**
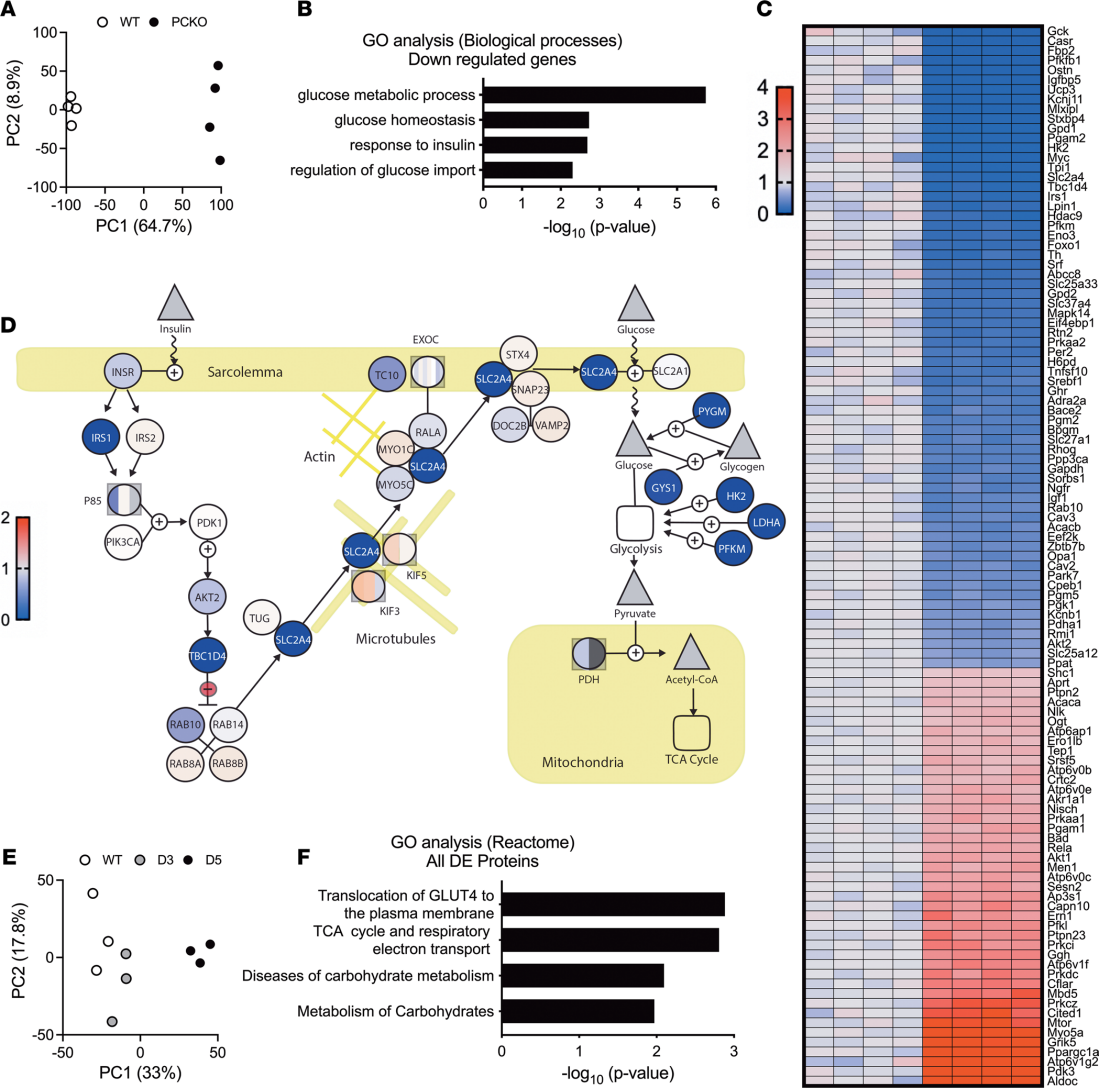
Loss of p300/CBP in skeletal muscle leads to the downregulation of insulin signaling and GLUT4 exocytic translocation genes and proteins. (**A**) Principal component analysis (PCA) plot for gene expression during microarray in WT and PCKO EDL muscles; WT/PCKO *n* = 4/4. (**B**) Gene Ontology (GO) biological processes terms relating to metabolism (FDR < 0.1) for significantly downregulated genes in PCKO versus WT muscles. (**C**) Expression profile (down [blue] and up [red]) for genes within biological processes GO categories from **B**. (**D**) Graphical representation of differentially expressed genes within the insulin signaling, GLUT4 exocytic translocation, and glucose metabolism pathways. (**E**) PCA plot for protein abundance during tandem mass tag mass spectrometry in WT, D3 PCKO, and D5 PCKO tibialis anterior muscle; WT/D3/D5 *n* = 3/3/3. (**F**) GO Reactome terms relating to metabolism (FDR < 0.1) for all differentially expressed proteins in PCKO D5 versus WT muscles.

For insight into why PCKO mice develop severe glucose intolerance and skeletal muscle insulin resistance, we mined publicly available gene microarray data from Gene Expression Omnibus (GEO accession: GSE141857) of skeletal muscle of D5 PCKO and WT mice, which we previously generated (27). Principal component analysis (PCA) demonstrated that PCKO mice had markedly altered mRNA expression of genes compared with WT mice, with the first principal component explaining 64.7% of the variance (Figure 3A). In line with our findings of severe glucose intolerance and skeletal muscle insulin resistance in PCKO mice, Gene Ontology (GO) enrichment analysis of downregulated genes (Supplemental Table 1) showed enrichment for biological processes important to glucose metabolic process, glucose homeostasis, response to insulin, and regulation of glucose import (Figure 3B). A heatmap of the genes comprising these GO terms reveals that more than 60% were downregulated in PCKO versus WT mice; in fact, across the downregulated genes, expression was reduced by an average of approximately 70% in PCKO mice (Figure 3C). Considering the robust impairment in insulin-stimulated glucose uptake in PCKO mice, we next superimposed a subset of this microarray data onto a graphical interpretation of the insulin signaling and GLUT4 trafficking pathway (Figure 3D and Supplemental Figure 4). This approach demonstrated that PCKO mice had a global reduction in the expression of genes relevant to skeletal muscle insulin signaling, GLUT4 exocytic translocation, and glucose metabolism.

### The abundance of insulin signaling and GLUT4 exocytic translocation proteins is substantially changed in PCKO muscle at D5 but not at D3.

While PCKO muscle had significant changes in mRNA expression of insulin signaling and GLUT4 trafficking genes compared with WT muscle, this cannot adequately explain the insulin-resistant phenotype in PCKO mice without considering the consequent changes in protein abundance. To address this, we mined publicly available quantitative proteomics data from PRoteomics IDEntifications Database (PRIDE project: PXD015694) in muscle from D3 and D5 PCKO, and D5 WT mice, which we previously generated (27). The PCA demonstrated that PCKO mice at D5 had a clearly different protein abundance profile compared with WT mice, whereas PCKO mice at D3 clustered closer to WT mice (Figure 3E). GO enrichment analysis for all differentially expressed proteins showed enrichment in the Reactome database for metabolic processes similar to the microarray data (Supplemental Table 2) and included “translocation of SLC2A4 (GLUT4) to the plasma membrane” (Figure 3F). Within this Reactome group (Reactome ID: R-HAS-1445148), most proteins had significantly reduced abundance at D5 (between 10% and 50% reduction; data not shown), while at D3 only TBC1D1 (~40%) and GLUT4 (~20%) had substantially reduced abundance compared with WT mice (data not shown).

Loss of insulin-stimulated glucose uptake in PCKO mice at D5 occurred in association with a downregulation in the gene expression and protein abundance of critical insulin signaling and GLUT4 exocytic translocation networks. Notably, however, the reduction in protein abundance of proteins important for insulin signaling to glucose transport was not proportional to the phenotype seen. For example, while there was a complete loss of insulin-stimulated glucose uptake in PCKO mice at D5, we saw only a 20% to 50% decrease in proteins involved in insulin signaling and GLUT4 trafficking, such as GLUT4, TBC1D1, and Rab10 (data not shown). Furthermore, PCKO mice were severely insulin resistant at D3, but their proteomics profile was relatively indifferent from WT mice. Considering the proteomics and microarray findings together, it is apparent that mRNA/protein changes did not fully account for the rapid loss of insulin-stimulated glucose uptake in skeletal muscle of PCKO mice. By extension, we interpret these findings to suggest that p300/CBP are potentially regulating insulin signaling to glucose transport via direct acetylation of target signaling proteins, as opposed to regulating it “indirectly” via their well-described effects on gene transcription.

### Acute inhibition of p300/CBP acetyltransferase activity in mouse skeletal muscle robustly impairs insulin sensitivity and responsiveness.

**Figure 4:**
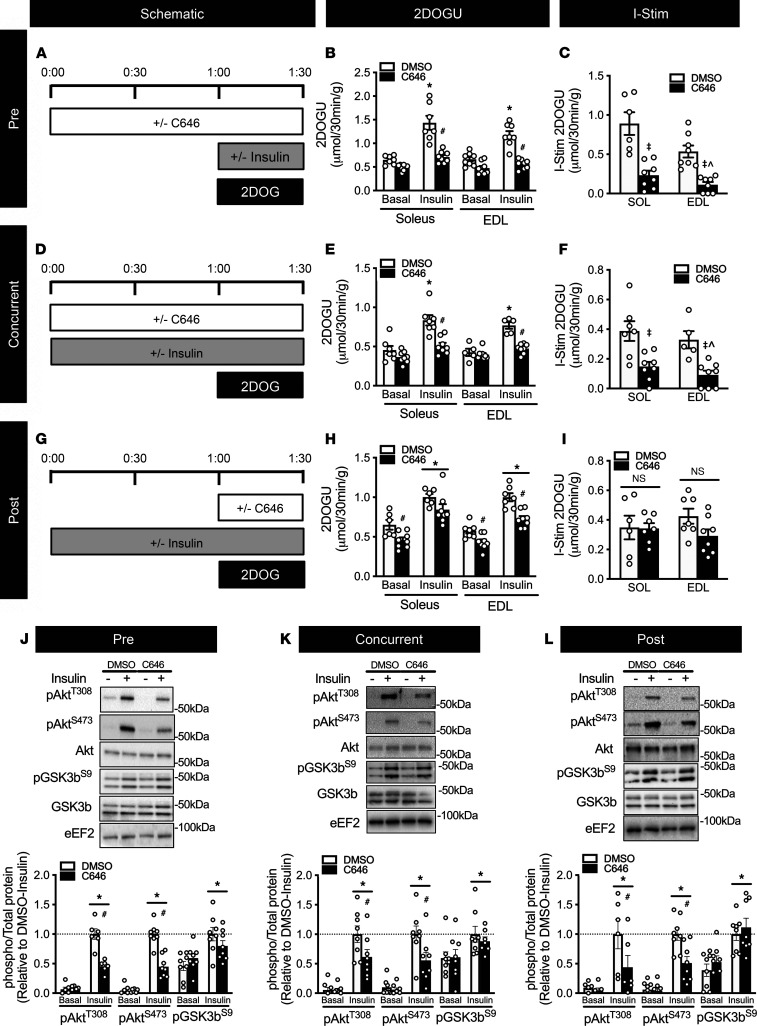
Acute inhibition of p300/CBP activity in skeletal muscle hinders insulin sensitivity independent of Akt activity. Schematic for (**A**) “Pre,” (**D**) “Concurrent,” and (**G**) “Post” C646 treatment experiments; 50 μM C646 was used for all experiments. Basal 2DOGU, insulin (0.36 nmol/L) 2DOGU, and insulin-stimulated 2DOGU (calculated as insulin 2DOGU – basal 2DOGU) in isolated soleus and EDL muscles from WT mice in (**B** and **C**) “Pre,” (**E** and **F**) “Concurrent,” and (**H** and **I**) “Post” experiments. (**J**–**L**) Representative images for pAkt^T308^, pAkt^S473^, total Akt, pGSK3b^S9^, and total GSK3b in basal and insulin-stimulated (- and +, respectively) EDL muscles from DMSO and C646 treatment groups for (**J**) “Pre,” (**K**) “Concurrent,” and (**L**) “Post” experiments; values are presented relative to DMSO-insulin. *, *P* < 0.05, 2-way ANOVA with Sidak’s multiple comparison versus basal within treatment. ^#^, *P* < 0.05 versus DMSO within insulin. For I-Stim, ^‡^, *P* < 0.05, 2-way ANOVA, main effect of diet. ^, *P* > 0.05, 1-sample *t* test versus “0.” DMSO/C646 *n* = 8/8. Data reported as mean ± SEM.

To test the possibility of “direct” regulation of insulin-stimulated glucose uptake by p300/CBP, we utilized a potent small molecule inhibitor of p300/CBP acetyltransferase activity, C646 (28). Specifically, we studied insulin action after treating muscles with C646 for 60 minutes or less, as we have done with other inhibitors (10). Since 60 minutes of C646 minimally affects transcriptional markers (28), observed changes after incubating with C646 for 60 minutes or less can be considered to be nontranscriptional. Remarkably, pretreatment of soleus and EDL with C646 for 1 hour (Figure 4A) blocked 2DOG uptake in the presence of insulin (Figure 4, B and C; and Supplemental Figure 5, A–C).

To confirm that effects seen with pretreatment with C646 were not due to transcriptional effects, we treated muscles concurrently with C646 and insulin (Figure 4D). Similar to C646 pretreatment, concurrent C646 treatment blocked 2DOG uptake in the presence of insulin (Figure 4, E and F). Considering that pre- and concurrent C646 treatment phenocopies PCKO mice, blocking insulin-stimulated glucose uptake, this supports the hypothesis that p300/CBP contribute to the direct regulation of insulin-stimulated glucose uptake. To further establish this, we reasoned that C646 treatment after muscles were incubated with insulin (Figure 4G) would not inhibit insulin-stimulated glucose uptake as activation of insulin signaling and stimulation and translocation of GLUT4 to the plasma membrane would have already taken place. Supporting this, unlike pre- and concurrent treatment with C646, the addition of C646 60 minutes after insulin stimulation did not affect insulin-stimulated 2DOG uptake in either soleus or EDL, as compared with DMSO (Figure 4, H and I).

In accordance with the insulin-stimulated 2DOG uptake data, insulin significantly increased phosphorylation of Akt^T308^, Akt^S473^, and its downstream target, GSK3b^S9^ (glycogen synthase kinase 3b), in the Pre (Figure 4J), Concurrent (Figure 4K), and Post (Figure 4L) experiments. Interestingly, however, while insulin-stimulated glucose uptake was reduced in only Pre and Concurrent treatment, C646 reduced insulin-stimulated Akt^T308^ and Akt^S473^ phosphorylation by approximately 50% in Pre, Concurrent, and Post C646 experiments. Critically, however, C646 treatment did not affect insulin-stimulated GSK3b^S9^ phosphorylation in any experiment condition, which suggests that the decline in insulin-stimulated Akt phosphorylation did not impair its downstream effects.

It is possible that the noted effects with C646 are the result of off-target effects. Thus, to further validate a potential role for p300/CBP in the direct regulation of insulin-stimulated glucose uptake by skeletal muscle, we studied a more recently validated and highly specific p300/CBP inhibitor, A-485 (29). The benefit of studying this compound is that its chemical structure is vastly different from C646, and as such, the possibility of the same off-target effects is low. Importantly, comparably to C646, preincubation (60 minutes) of both soleus and EDL with A-485 (Supplemental Figure 6A) blocked insulin-stimulated glucose uptake such that it was not significantly different from basal (Supplemental Figure 6, B–D).

Last, as our experiments investigated the role of p300/CBP on insulin sensitivity with a physiological concentration of insulin, it is possible that a supraphysiological dose of insulin could overcome the phenotypes seen. Interestingly, however, a supraphysiological dose of insulin could not rescue the skeletal muscle insulin resistance in PCKO mice (Supplemental Figure 7, A and B) or C646 treatment (Supplemental Figure 7C).

Overall, these experiments support a role for p300/CBP acetyltransferase activity in the direct regulation of insulin-stimulated glucose uptake by skeletal muscle. Furthermore, considering insulin-stimulated Akt activity was not reduced by C646 treatment (as indicated by no effect on insulin-stimulated GSK3b^S9^ phosphorylation), these data suggest that p300/CBP act at a step distal to Akt in the regulation of insulin-stimulated glucose uptake.

### Insulin stimulation increases lysine acetylation in muscle, glucose uptake in L6 and human skeletal muscle cells, and 3T3-L1 adipocytes in a p300/CBP-dependent manner.

**Figure 5:**
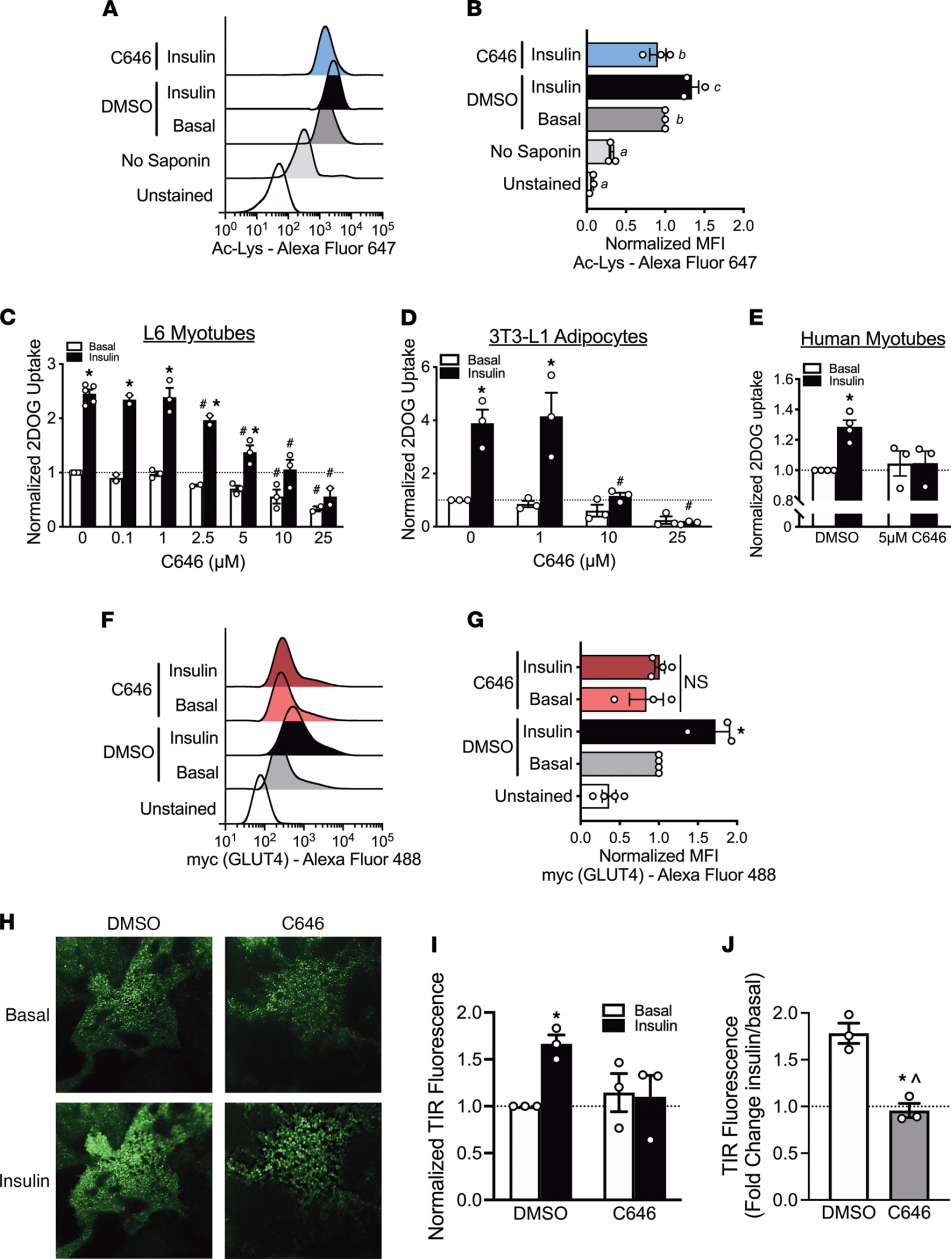
p300/CBP activity is required for insulin-stimulated acetylation and GLUT4 trafficking. (**A**) Representative experiment and (**B**) normalized mean fluorescence intensity for total acetylated proteins (Ac-Lys) in L6 myoblasts in unstained, stained without saponin, DMSO, 100 nM insulin, or insulin cotreated with 10 μM C646 conditions, as analyzed by flow cytometry. Different letters signify *P* < 0.05 1-way ANOVA, Tukey’s multiple-comparison test. All results are representative of 3 independent experiments. Normalized basal and insulin (100 nM) 2DOGU in (**C**) L6 myotubes, (**D**) 3T3-L1 adipocytes, and (**E**) human skeletal muscle myotubes pretreated for 1 hour with DMSO or C646. *, *P* < 0.05 2-way ANOVA with Sidak’s multiple comparison versus basal within C646 concentration. ^#^, *P* < 0.05 2-way ANOVA with Sidak’s multiple comparison versus DMSO within insulin treatment. All results are representative of 3 independent experiments. (**F**) Representative experiment and (**G**) normalized mean fluorescence intensity for plasma membrane localized GLUT4 in L6-GLUT4-myc myoblasts in unstained, and with or without 25 μM C646 or 100 nM insulin, as analyzed by flow cytometry. *, *P* < 0.05 2-way ANOVA with Sidak’s multiple comparison versus basal within treatment. Results are representative of 4 independent experiments. (**H**) Representative total internal reflection fluorescence imaging, using a 60× oil objective (n.a. 1.45), of Myc7-Glut4-eGFP in 3T3-L1 adipocytes 20 minutes after insulin administration and (**I** and **J**) its normalized quantitation. (**I**) *, *P* < 0.05 2-way ANOVA with Sidak’s multiple comparison versus basal within treatment. (**J**) *, *P* < 0.05 *t* test versus DMSO. ^, *P* > 0.05 1-sample *t* test versus “1.” Results are representative of 3 independent experiments with 6–11 cells per experiment. Data reported as mean ± SEM.

A key premise of this study is that p300/CBP acetyltransferase activity is integral to insulin-stimulated glucose uptake. By extension, we would expect p300/CBP to be located within the cytosol for insulin stimulation to increase protein acetylation of cytosolic insulin signaling proteins, as it does with phosphorylation. Indeed, CBP is found in both nuclear and cytosolic fractions of skeletal muscle lysate as assessed by subcellular fractionation (Supplemental Figure 8). We then assessed single-cell changes in acetylation in L6 myoblast cells using flow cytometry with insulin and C646 treatment. Insulin treatment of L6 myoblasts significantly increased pan-protein lysine acetylation, with this increase in acetylation blocked by p300/CBP inhibition with C646 (Figure 5, A and B). Confirming the L6 data, insulin treatment of mouse skeletal muscle significantly increased lysine acetylation as measured in immunoprecipitants using a pan–acetyl-lysine antibody (Supplemental Figure 9).

For mechanistic insight into direct regulation of insulin-stimulated glucose uptake by p300/CBP, we employed common cell-based model systems: L6 myotubes and 3T3-L1 adipocytes. We utilized 3T3-L1 adipocytes as a model system, alongside L6 myotubes, because the mechanism of insulin-stimulated GLUT4 exocytic translocation and glucose uptake in adipocytes is similar to muscle (1). Our rationale is that if p300/CBP activity are fundamentally required for insulin-stimulated GLUT4 exocytic translocation and glucose uptake, then their importance would be conserved in adipocytes and skeletal muscle. Thus, insulin increased glucose uptake above basal (i.e., no insulin) in L6 myotubes and 3T3-L1 adipocytes by approximately 2.5- and approximately 4-fold, respectively (Figure 5, C and D). In both cell types, C646 pretreatment (1 hour) dose-dependently led to inhibition of insulin-stimulated glucose uptake (Figure 5, C and D); notably, C646 pretreatment also dose-dependently inhibited basal glucose uptake in both cell types (Figure 5, C and D), albeit at higher concentrations. For translational insight and because these are immortalized cell lines, we also studied primary human skeletal muscle cells. As in L6 myotubes and 3T3-L1 adipocytes, C646 pretreatment inhibited insulin-stimulated glucose uptake in human skeletal muscle cells; basal glucose uptake was not affected by C646 treatment (Figure 5E).

### p300/CBP activity is required for insulin-stimulated GLUT4 trafficking and fusion to the plasma membrane.

As insulin-stimulated glucose uptake is tightly coupled to the abundance of GLUT4 that is fused with the plasma membrane (30), we reasoned that p300/CBP must be modulating GLUT4 trafficking to, and/or fusion with, the plasma membrane. To this end, we first determined the effect of p300/CBP inhibition on insulin-stimulated fusion of GLUT4 to the plasma membrane using a flow cytometry approach. As expected, insulin increased plasma membrane–fused GLUT4 in L6 myoblasts by approximately 1.7-fold (Figure 5, F and G). Significantly, in line with its effects on insulin-stimulated acetylation and 2DOG uptake, C646 pretreatment inhibited insulin-stimulated GLUT4 fusion to the plasma membrane (Figure 5, F and G).

After determining p300/CBP activity is required for insulin-stimulated GLUT4 fusion, we used total internal reflection fluorescence (TIRF) microscopy in 3T3-L1 adipocytes expressing GLUT4-eGFP to visualize GLUT4 dynamics within approximately 100–200 nm of the plasma membrane. Treatment of 3T3-L1 adipocytes with insulin significantly increased the intensity of GLUT4-eGFP found within the TIRF zone (Figure 5, H–J). Remarkably, pretreatment (1 hour) with C646 blocked this increase (Figure 5, H–J). From these data, single molecule tracking (Supplemental Figure 10A) revealed that while insulin decreased the diffusion coefficient and distance traveled by a GLUT4 molecule in DMSO-treated cells, this effect was prevented by C646 pretreatment (Supplemental Figure 10, B and C). Furthermore, C646 treatment reduced the diffusion coefficient and distance traveled in the basal state (no insulin). These observations are further represented by mean square displacement curves of representative GLUT4 molecules for each treatment, demonstrating that while basal cells had GLUT4 molecules moving freely via diffusion (following a dotted line), insulin or C646 treatment constrained the movement of GLUT4 molecules (Supplemental Figure 10D).

## Discussion

While p300 and CBP are global transcriptional regulators in skeletal muscle (12), the regulatory crosstalk between them and cytosolic insulin signaling proteins (15–18) suggests they could directly regulate insulin action. Addressing this, here we report that loss of both p300 and CBP acetyltransferase activity causes a complete loss of insulin-stimulated glucose uptake in skeletal muscle and adipocytes and does so via inhibition of insulin-stimulated GLUT4 exocytic translocation. Together, these data establish p300/CBP as direct, fundamental regulators of insulin-stimulated glucose uptake in skeletal muscle and adipocytes.

A phosphorylation-based model for insulin-stimulated glucose transport has been the current paradigm for over 30 years (1, 2). Within this framework, particular kinases have been established as fundamental to insulin action; for example, PI3K and Akt2 are required for insulin-stimulated glucose uptake independent of muscle fiber type, tissue type (i.e. muscle and adipocytes), or sex (1, 2). In the present study, we describe a similarly broad conservation of importance for p300/CBP and acetylation in regulating insulin action. Supporting this, first, we demonstrate that insulin stimulation can induce both glucose uptake and the acetylation of proteins within skeletal muscle and that both these processes are p300/CBP dependent. Our data imply that acetylation has a level of regulatory complexity similar to phosphorylation, where insulin-stimulated glucose uptake is dependent on a specific kinase (i.e., PI3K, Akt2) and acetyltransferase (i.e., p300/CBP). While these effects may be, in part, due to inhibition of deacetylase activity, it is important to note that pharmacologically inhibiting protein deacetylases, which nondiscriminatorily increases protein acetylation, does not impact insulin-stimulated glucose uptake in L6 myotubes or mouse muscle (10). Similarly, as it relates to the sirtuins (SIRTs) SIRT1 and SIRT3, which are the most studied deacetylases and abundant SIRTs in skeletal muscle, knockout of SIRT1 or SIRT3 in mice does not impair insulin-stimulated plasma membrane GLUT4 abundance or insulin sensitivity in skeletal muscle (9, 31), nor does overexpression of SIRT1 enhance it (32). Moreover, SIRT2 inhibition in C2C12 myotubes (33) or 3T3-L1 adipocytes (34) does not alter insulin action. Another important consideration for the role of deacetylases in our model, however, is the reversal of insulin-stimulated glucose uptake, akin to phosphatases for phosphorylation; this could occur, in part, by effects on GLUT4 endocytosis as deacetylases have been shown to regulate endocytosis in other systems (35, 36).

As orthologs, p300 and CBP have high sequence homology and highly compensatory and redundant functions (12). Thus, it is not unexpected that we found that inhibition of *both* p300 and CBP activity was necessary to see effects on insulin-stimulated glucose uptake; for example, in the single-allele rescue experiment (i.e., CBP KO/p300 HZ and p300 KO/CBP HZ mice), the insulin-stimulated glucose uptake by skeletal muscle and glucose tolerance defects in PCKO mice were completely normalized to WT values. This high degree of redundancy further supports the fundamental role of p300/CBP in regulating insulin-stimulated glucose uptake. Second, p300/CBP activity is required for insulin-stimulated glucose uptake not only in skeletal muscle but also in adipocytes, which have a similarly conserved signaling pathway (1). While it is possible that inhibition of either p300 or CBP alone may recapitulate these findings in adipocytes, it is unlikely as single knockout of p300 or CBP in adipose tissue (37) does not affect glucose tolerance, which is similar to the phenotype found in mice with single knockout of p300 or CBP in skeletal muscle (24). Furthermore, p300/CBP are required for insulin-stimulated glucose uptake regardless of fiber type, sex, or body weight. PCKO mice, in fact, have reduced body weight, which is typically associated with improved skeletal muscle insulin sensitivity (9, 24). Last, demonstrating translational significance, the requirement of p300/CBP activity for insulin-stimulated glucose uptake is conserved across species (mouse muscle, rat L6 myotubes, and human skeletal muscle myotubes) as well as insulin doses. In insulin-resistant humans, the dose response curve for insulin-stimulated whole-body glucose disposal (which is driven by skeletal muscle) is shifted both to the right (reduced insulin sensitivity) and downward (reduced insulin responsiveness) (38, 39). While most studies test for only one of either insulin sensitivity or responsiveness (40), herein we demonstrate a reduction in both with inhibition of p300/CBP activity, which correlates with the pathophysiology of human insulin resistance. Hence, we establish p300/CBP jointly, together with acetylation, as potentially novel fundamental regulators of insulin-stimulated glucose uptake alongside the canonical phosphorylation-based system.

p300/CBP are critical regulators of transcription (12). As such, phenotypes that manifest from modulating p300/CBP are generally thought to be due to transcriptional mechanisms. For example, p300/CBP regulate muscle integrity and differentiation in human primary myoblasts and zebrafish (41), as well as adipose tissue development in mice, all via transcriptional control. In line with this, PCA plots of the microarray data in PCKO versus WT mice demonstrate that these muscles are transcriptionally distinct, including a drastic downregulation of mRNA expression of critical insulin signaling and GLUT4 exocytic translocation genes. Thus, the severe impairments in insulin-stimulated glucose uptake in PCKO mice could be partly due to transcriptional changes. However, because p300/CBP have a strong cytosolic presence in mesodermal origin cell types (14, 42–44) (including in muscle shown here) and can acetylate various proteins key to insulin signaling and GLUT4 exocytic translocation (14–18), it is difficult to know whether the phenotype of PCKO mice is due to p300/CBP regulating insulin action via transcriptional or nontranscriptional mechanisms. Our experiments using 2 structurally distinct compounds (i.e., C646 and A-485) to briefly inhibit p300/CBP acetyltransferase activity address this issue and clearly demonstrate that p300/CBP can directly (i.e., independent of transcription) regulate insulin-stimulated glucose uptake, be it in skeletal muscle (including primary human and L6 myotubes and mature mouse skeletal muscle) or adipocytes.

Proximal insulin signaling, and particularly Akt2 activity, is a major determinant of insulin action (1, 45). Given that p300/CBP appear to be acting in a direct manner in the regulation of insulin-stimulated glucose uptake, we first investigated the impact of the loss of p300/CBP activity on proximal insulin signaling. In PCKO mice, insulin-stimulated Akt phosphorylation was identical to WT mice, yet insulin-stimulated glucose uptake was severely impaired. While insulin-stimulated glucose uptake was similarly impaired with acute C646 treatment, phosphorylation of Akt^S473^ was reduced by 50%. However, this was not accompanied by a reduction in insulin-mediated phosphorylation of GSK3b^S9^, a direct substrate of Akt. Furthermore, despite this reduction in Akt phosphorylation, insulin-stimulated glucose uptake was not reduced when C646 was given after insulin treatment, even though phosphorylation of Akt^S473^ was reduced by 50%. The reason for this disconnect could be due to the fact that only a minimal amount of Akt activity is required for downstream effects on phosphorylation of GSK3b^S9^ and maximum insulin-stimulated glucose uptake (46, 47). Taken together, as a 50% reduction in Akt phosphorylation does not impact insulin-stimulated glucose uptake, and both genetic and pharmacological inhibition of p300/CBP activity block insulin-stimulated glucose uptake, our data suggest that p300/CBP regulate skeletal muscle insulin sensitivity downstream of Akt and the proximal insulin signaling pathway.

While the proteins involved in proximal insulin signaling are well established, the identity of those important for GLUT4 trafficking and exocytosis are less so; however, insulin-stimulated exocytic translocation of GLUT4 is known to occur via 3 distinct steps: translocation, tethering, and fusion (1). Utilizing flow cytometry in L6 myoblasts and TIRF microscopy in 3T3-L1 adipocytes, we demonstrate that p300/CBP activity is required not only for insulin-stimulated GLUT4 fusion to the plasma membrane but also for insulin-stimulated GLUT4 movement into the TIRF zone. While this does not rule out p300/CBP activity being necessary for the fusion step, it does indicate that there is a more proximal step that p300/CBP activity is critical for in the GLUT4 itinerary. Interestingly, inhibition of p300/CBP activity also reduced GLUT4 movement within the TIRF zone when insulin was not present, further indicating the importance of p300/CBP activity toward the function of GLUT4 trafficking. Since the TIRF zone includes machinery important for the tethering and fusion steps of GLUT4 trafficking, but not translocation (1, 48), this indicates that p300/CBP are likely acting proximally at the translocation step of GLUT4 exocytic translocation.

The translocation step of GLUT4 trafficking involves the release of GLUT4 storage vesicles from the perinuclear region, allowing for travel toward the plasma membrane first along microtubules via kinesins, followed by cortical actin via myosins (48). One portion of the translocation step that p300/CBP is not likely to be acting upon is cortical actin and myosin proteins. p300/CBP can acetylate Rho guanine nucleotide dissociation inhibitor α, which induces actin polymerization (49), and actin polymerization is critical for insulin-stimulated glucose uptake in skeletal muscle (50). Importantly, however, inhibition of actin polymerization still allows for GLUT4 to reach the TIRF zone in 3T3-L1 adipocytes (51) and does not constrict the movement of GLUT4 within the TIRF zone (52) and therefore would not fit with our data. One point of regulation for p300/CBP may be in the release of GLUT4 from the perinuclear region via tether containing UBX domain for GLUT4 (TUG). The acetylation of TUG promotes GLUT4 accumulation at the plasma membrane of 3T3-L1 adipocytes (11). Therefore, a loss of p300/CBP activity (and subsequent TUG acetylation) could lead to reduced GLUT4 release and result in a loss of insulin-stimulated glucose uptake. Notably, while SIRT2 was identified as the deacetylase for TUG (11), the acetyltransferase responsible for TUG acetylation is currently unknown. Importantly, however, TUG mutants or SIRT2 overexpression did not abolish insulin-stimulated GLUT4 translocation as in p300/CBP inhibition, but rather decreased it (~25%–50%) (11), which suggests that another protein or multiple proteins are important. Another point of regulation for p300/CBP could be the microtubule network. Both tubulin polymerization (53) and kinesin function (54) are critical for insulin-stimulated GLUT4 exocytic translocation and glucose uptake in adipocytes. Furthermore, inhibition of tubulin polymerization, similar to p300/CBP inhibition, blocks insulin-stimulated glucose uptake, movement of GLUT4 into the TIRF zone, as well as basal and insulin-stimulated GLUT4 mobility within the TIRF zone in 3T3-L1 adipocytes (53). Notably, C646 can inhibit polymerization of tubulin at concentrations as low as 25 μM (55), and therefore it is possible that the phenotype seen could be due to off-target effects of C646. However, the study by Shrimp et al. utilized a C646 clickable analog “C646-yne,” and experiments were done in vitro and not in muscle or adipocyte systems. Furthermore, we demonstrate a dose-dependent response of our phenotype with concentrations of C646 as low as 5 μM in L6 myotubes, 3T3-L1 adipocytes, and mouse skeletal muscle and confirm our results with an alternate p300/CBP inhibitor, A-485. Importantly, p300 can regulate the acetylation of tubulin via regulation of SIRT2 and histone deacetylase 6 (HDAC6) activity (56); acetylation of tubulin promotes kinesin binding and motility (57, 58), which could enhance GLUT4 vesicle translocation dynamics. Thus, it is possible that p300/CBP regulate insulin action via the action of HDAC6 and SIRT2 on tubulin; however, knockout of these proteins does not appear to affect insulin-stimulated glucose uptake in a direct manner. For example, while adipocyte-specific knockout of HDAC6 in mice causes glucose intolerance and skeletal muscle insulin resistance during a hyperinsulinemic-euglycemic clamp (59), this is attributed to indirect effects of increased lipid accumulation via transcription and not a direct effect on insulin signaling or GLUT4 exocytic translocation proteins. Furthermore, mice with a knockout of SIRT2 are not insulin resistant (60); thus p300/CBP regulation of tubulin polymerization and subsequent insulin action, at least via SIRT2 and/or HDAC6, appears unlikely. Thus, while p300/CBP activity is critical for the translocation step of GLUT4 exocytic translocation, the subject of future research will include whether p300/CBP are indeed the direct targets of these proteins in skeletal muscle and within the context of insulin stimulation.

In summary, we establish p300/CBP as fundamental, direct regulators of insulin-stimulated glucose uptake in both skeletal muscle and adipocytes and demonstrate that this regulation occurs via effects on GLUT4 exocytotic translocation. It will be of high interest in future studies to interrogate how the acetylation of proteins within the GLUT4 exocytic translocation pathway could affect insulin sensitivity and GLUT4 translocation dynamics in the context of insulin stimulation, particularly in the context of insulin-resistant (e.g., type 2 diabetes, obesity) and insulin-sensitized (e.g., calorie restriction, exercise) states.

## Methods

### Mouse models.

Studies were conducted in male and female mice on a C57BL/6J background and housed in a conventional facility with 12-hour light/12-hour dark cycle. Inducible, skeletal muscle–specific, p300 and CBP double-knockout mice (PCKO), as well as mice with only 1 allele of either p300 (CBP KO/p300 HZ) or CBP (p300 KO/CBP HZ) in skeletal muscle, have been described previously (27). Respective floxed, but Cre-negative, littermates were used as experimental controls for the mouse models; collectively, these mice are referred to as WT. At 13–15 weeks of age, all mice were orally gavaged with tamoxifen (2 mg) (Toronto Research Chemicals) for up to 5 consecutive days to activate skeletal muscle–specific Cre recombinase. For studies in PCKO, CBP KO/p300 HZ, p300 KO/CBP HZ, and WT mice, tissue collection, OGTT, and ex vivo 2DOG uptake assays were performed at 1, 3, or 5 days after initiating tamoxifen dosing, as indicated. Inhibitor-based studies were conducted in male or female C57BL/6J (Jackson Laboratory, stock 05304) mice at 13 weeks of age, as indicated.

### OGTT.

Fasted (4 hours) mice were orally gavaged with dextrose (2 g/kg), and blood glucose concentration was measured via the tail vein at 0 (before gavage), 20, 30, 45, 60, 90, and 120 minutes after gavage using a handheld glucose meter (Ascensia Contour, Bayer HealthCare). AUC was calculated using Prism 8 (GraphPad Software) with 0 mg/dL used as the baseline.

### Skeletal muscle 2DOG uptake in p300/CBP KO mouse lines.

Basal and insulin-stimulated 2DOG uptake was measured in isolated and paired soleus and EDL muscles of transgenic mice, as previously described (9, 24). Muscles were incubated with insulin at a physiological concentration of 0.36 nM for all experiments other than Supplemental Figure 7, which used a supraphysiological concentration of 6 nM.

### Skeletal muscle 2DOG uptake with C646.

For inhibitor-based studies, some modifications were made (Figure 4, A, E, and I). For the Pre experiment, paired soleus and EDL muscles were placed into a preincubation solution including 0.2% DMSO or 50 μM C646 (Selleckchem, S7152) for 1 hour. Subsequently, muscles were transferred into an incubation solution that included [^3^H]2DOG and [^14^C]mannitol for 30 minutes, with muscles from one side being exposed to insulin (0.36 nM for all experiments other than Supplemental Figure 7, where 6 nM was used) and the contralateral side exposed to no insulin (Basal). During this 30-minute period, muscles continued to be incubated with DMSO or C646. For the Concurrent experiment, paired soleus and EDL muscles were placed into a preincubation solution including DMSO or 50 μM C646, with muscles from one side being exposed to insulin (0.36 nM) and the contralateral side exposed to no insulin, for 1 hour. Subsequently, muscles were transferred into an incubation solution that included [^3^H]2DOG and [^14^C]mannitol for 30 minutes, during which muscles continued to be incubated with or without C646 and with or without insulin. For the Post experiment, paired soleus and EDL muscles were placed into a preincubation solution with muscles from one side being exposed to insulin (0.36 nM) and the contralateral side exposed to no insulin, for 1 hour. Subsequently, muscles were transferred into an incubation solution that included [^3^H]2DOG and [^14^C]mannitol for 30 minutes, with muscles being treated with either DMSO or 50 μM C646. During this 30-minute period, muscles continued to be incubated with or without insulin.

### Skeletal muscle 2DOG uptake with A-485.

Paired soleus and EDL muscles from 13-week-old female mice were placed into a preincubation solution including 0.2% DMSO or 50 μM A-485 (MedChem Express LLC, HY-107455; DMSO final concentration also 0.2%) for 1 hour. Subsequently, muscles were transferred into an incubation solution that included [^3^H]2DOG and [^14^C]mannitol for 30 minutes, with muscles from one side being exposed to insulin (0.36 nM) and the contralateral side exposed to no insulin (Basal). During this 30-minute period, muscles continued to be incubated with DMSO or A-485 (Supplemental Figure 6A).

### Tissue collection.

Tissues were excised from fasted (4 hours), anesthetized mice; rapidly washed in 0.9% saline; blotted dry; and frozen in liquid nitrogen. Tissues were stored at –80°C for subsequent analysis.

### Plasma insulin concentration.

Plasma insulin was analyzed using an ELISA kit, per the manufacturer’s instructions (80-INSMS-E-01; ALPCO Diagnostics).

### Immunoblotting.

Immunoblotting was performed, as previously described (24). All antibodies were used at a dilution of 1:1000, unless stated otherwise: Akt (Cell Signaling Technology 9272B), phosphorylated (p) Akt^T308^ (1:500; Cell Signaling Technology 9275), pAkt^S473^ (Cell Signaling Technology 4058), glycogen synthase kinase 3 α/β (GSK3a/b; Cell Signaling Technology 5676), pGSK3a/b^S21/9^ (Cell Signaling Technology 9331), Lamin A/C (Cell Signaling Technology 4777), CBP (Cell Signaling Technology 7389), and eukaryotic elongation factor 2 (eEF2; Cell Signaling Technology 2332). Densitometric analysis of immunoblots was performed using Image Lab (Bio-Rad). Phosphorylated protein abundance was normalized to respective total protein abundance. Full, uncut gels are available online as supplemental material.

### Microarray.

Microarray data are stored and publicly available on the GEO database (GEO accession: GSE141857). The sorted list of genes was analyzed for overrepresented biological processes defined by the Gene Ontology Consortium, using conditional gene ontology analysis (61, 62), with a primary focus on biological processes related to glucose metabolism, insulin signaling, and GLUT4 trafficking.

### Insulin signaling/GLUT4 exocytic translocation network map.

The network map was based on published transcriptional networks (1, 63). Monochrome circles represent expression of individual genes, color-coded by fold change (KO/WT), with red shades indicating upregulation and blue shades indicating downregulation. Gene labels are Entrez gene symbols. Multicolored circles within a grayed box indicate complexes (detailed in Supplemental Figure 4), with the expression of each component gene indicated by ordered stripes. White squares with rounded edges indicate modules or functions and gray triangles indicate nonprotein molecules. Connectivity between proteins encoded by specified genes is indicated by the following symbols: plus in white circle (positive), minus in red circle (negative), straight arrow (A proceeds to B), and curved arrow (translocation of A). Proteins encoded by specified genes are drawn in their approximate anatomical location relative to cellular structures (yellow).

### Tandem mass tag mass spectrometry.

Proteomics data are stored and publicly available on the PRIDE database (project: PXD015694). The sorted list of proteins was analyzed for enriched pathways using the Reactome database (64), with a primary focus on reactomes related to glucose metabolism, insulin signaling, and GLUT4 trafficking.

### L6 cell culture conditions.

Rat skeletal muscle–derived L6 myoblast cells and L6 myoblasts expressing GLUT4 with an exofacial myc epitope tag (L6-GLUT4-myc; both from Kerafast) were cultured in monolayers on 100 mm cell culture treated dishes, using Minimum Essential Media *a* (MEM*a;* Gibco, 12571071) supplemented with 10% fetal bovine serum (FBS; VWR, 89510-186), at an atmosphere of 5% CO_2_ at 37°C. Media for L6 myoblasts were replenished every 48 hours. L6 myoblasts were maintained by trypsinization of cultures at 70% to 80% confluence and were only assayed up to passage 15. Myoblasts were seeded at a density of 2 × 10^4^ cells/well in 24-well plates for glucose uptake studies. After reaching confluence, myoblasts were differentiated into myotubes in MEM*a* supplemented with 2% FBS for 4 days.

### Subcellular fractionation.

Subcellular fractionation was done using the NE-PER nuclear and cytoplasmic extraction kit, per the manufacturer’s instructions (catalog number 78833; Thermo Fisher Scientific).

### Gastrocnemius insulin stimulation for immunoprecipitation and silver staining of acetylated proteins.

Male C57BL/6J (Jackson Laboratory, stock 05304) mice at 13 weeks of age were anesthetized, and the right gastrocnemius muscle was dissected. A total of 1 U/kg insulin was then injected into the inferior vena cava, and after 2 minutes the left gastrocnemius muscle was dissected. Tissue was collected and homogenized as described above. Immunoprecipitation with anti–acetyl-lysine (AAC-01; Cytoskeleton) and silver stain (Pierce Silver Stain kit 24612; Thermo Fisher Scientific) were conducted and analyzed as described previously (24).

### Flow cytometry.

L6 myoblasts were serum-starved, in suspension, for 3 hours in 0.5% fatty acid–free BSA in MEM*a*. For pan-acetylation assays, after serum-starving, L6 myoblasts were suspended in HEPES-buffered Krebs-Ringer bicarbonate buffer (HKRB; 10 mM HEPES, 4.8 mM KCl, 1 mM CaCl_2_, 1 mM MgCl_2_, and 0.5% fatty acid–free BSA in Dulbecco’s phosphate-buffered saline [DPBS]) with or without 100 nM insulin, with DMSO or 10 μM C646 for 1 hour. L6 myoblasts were fixed in 2% paraformaldehyde at room temperature (RT) for 30 minutes, washed twice with DPBS, permeabilized with 0.5% saponin at RT for 30 minutes, and incubated with Alexa Fluor 647–labeled acetylated lysine (1:20 dilution; 623405, BioLegend) at RT for 30 minutes. For the GLUT4 fusion assay, after serum starvation L6-GLUT4-myc myoblasts were suspended in HKRB with DMSO or 10 μM C646 for 1 hour. Subsequently, cells were treated with or without 100 nM insulin for 30 minutes. L6 myoblasts were fixed in 2% paraformaldehyde at RT for 30 minutes, washed twice with DPBS, and incubated with Alexa Fluor 488–labeled myc antibody (1:50 dilution; 2279, Cell Signaling Technology) at RT for 30 minutes. An equal concentration of DMSO was used in non–C646-treated cells. For all flow cytometry experiments, at least 5000 cells were counted per condition. Flow cytometric acquisitions were performed using a ZE5 Cell Analyzer (Bio-Rad).

### L6 myotube 2DOG uptake.

L6 myotubes were serum-starved for 3 hours in 0.5% fatty acid–free BSA in MEM*a* followed by 1 hour in HKRB with DMSO or varying concentrations of C646, then subsequently stimulated with or without insulin (100 nM) for 40 minutes. For the last 10 minutes of the 40-minute insulin stimulation, 1 mM [^3^H]2DOG was present. The reaction was terminated by quick aspiration, and cells were washed twice with ice-cold DPBS. Cells were dissolved with 1 M NaOH and neutralized with 1 M HCl. The radioactivity of the cell lysates was measured using a liquid scintillation counter. Radioactivity was normalized to the protein concentration of the samples, using the Pierce BCA protein assay kit (23225; Thermo Fisher Scientific). Data were normalized to cells treated with DMSO and no insulin, within each experiment.

### Human skeletal muscle cell 2DOG uptake.

Human skeletal muscle cells were isolated, grown, differentiated, and assayed for glucose transport as previously described (65), with some modifications. Cells were first pretreated with or without 5 μM C646 for 1 hour prior to the addition of insulin (100 nM) for 30 minutes. An equal concentration of DMSO was used in non–C646-treated cells. Data were normalized to cells treated with DMSO and no insulin, within each experiment.

### 3T3-L1 cell culture conditions.

3T3-L1 fibroblasts (ATCC) and 3T3-L1 fibroblasts constitutively expressing myc7-GLUT4-eGFP were cultured and differentiated into adipocytes as previously described (66). Briefly, 3T3-L1 fibroblasts were grown on 100 mm cell culture treated dishes, using Dulbecco’s modified Eagle medium (DMEM; Gibco, 11995073) supplemented with 10% newborn calf serum (Gibco, 16010159), at an atmosphere of 5% CO_2_ at 37°C. For differentiation into adipocytes, fibroblasts were cultured for 3–5 days after reaching confluence with changes of medium every 48 hours. Subsequently, cells were cultured for 3 days in DMEM containing 10% FBS, 1 μg/mL insulin (MilliporeSigma I-5523), 0.25 μM dexamethasone (MilliporeSigma D-1756), and 0.5 mM methylisobutylxanthine (MilliporeSigma I-5879), followed by 2 days in DMEM containing 10% FBS and 1 μg/mL insulin. After differentiation, adipocytes were maintained in DMEM supplemented with 10% FBS.

### 3T3-L1 adipocyte 2DOG uptake.

Basal and insulin-stimulated glucose uptake in 3T3-L1 adipocytes was assessed as previously described (66), with some modifications. Cells were first pretreated with or without varying concentrations of C646 for 1 hour prior to the addition of insulin (100 nM). An equal concentration of DMSO was used in non–C646-treated cells. Data were normalized to cells treated with DMSO and no insulin, within each experiment.

### TIRF microscopy.

3T3-L1 adipocytes constitutively expressing Myc7-Glut4-EGFP were pretreated with 25 μM C646 or DMSO for 1 hour in imaging buffer (sterile filtered deionized water with 129 mM NaCl, 4.7 mM KCl, 1.2 mM KH_2_PO_4_, 5 mM NaHCO_3_, 2.5 mM CaCl_2_, 1.2 mM MgCl_2_, 10 mM HEPES, 3 mM glucose, and 0.1% BSA). Cells were imaged before and after 20 minutes of 100 nM insulin stimulation. Cells were imaged with an Applied Precision OMX microscope equipped with a ring TIRF system, a 60× oil objective (n.a. 1.45), and DeltaVision OMX software. Excitation was performed with a 488 nm laser. During imaging, cells were enclosed in a 37°C heated chamber with 5% CO_2_. ImageJ (NIH) was used to process and quantify images to calculate TIRF, diffusion coefficients, and distance traveled for individual GLUT4 molecules, as described previously (67).

### Statistics.

For data other than microarray and proteomics analysis (see ref. 27), an unpaired 2-tailed *t* test or 1-way or 2-way ANOVA (using repeated measurement where appropriate), followed by Sidak’s or Tukey’s post hoc test, was used. Significance was set at *P* < 0.05. When there were no significant differences among the respective WT mice (e.g., D1 vs. D3 vs. D5 or CBP KO/p300 HZ vs. p300 KO/CBP HZ vs. PCKO), data were pooled together, as noted. Statistical analyses were performed using Prism 8 (GraphPad Software). All data are expressed as mean ± SEM.

### Study approval.

Procedures were carried out with the approval of, and in accordance with, the Animal Care Program and Institutional Animal Care and Use Committee at the University of California, San Diego.

## Author contributions

SS, CEM, and VFM were responsible for the conception and design of the study. SS, VFM, SAL, AS, and DB were responsible for the analysis and interpretation of the data. VFM was responsible for the design and drafting of the manuscript and SS revised the manuscript critically. KS, CWH, OK, TPC, BH, GAM, AP, LLD, RRH, JEA, ARS, JEP, CH, and CEM contributed to analysis and interpretation of data and critical revision of the manuscript. All authors gave final approval.

## Supplementary Material

Supplemental data

Supplemental tables 1-2

## Version 1 11/23/2021

In-Press Preview

## Version 2 01/11/2022

Electronic publication
