# Supplementary material for: p300 or CBP is required for insulin-stimulated glucose uptake in skeletal muscle and adipocytes
Source: JCI Insight. 2022 Jan 11;7(1):e141344. doi: 10.1172/jci.insight.141344 (PMC8765050; doi:10.1172/jci.insight.141344)
Supplement: Supplemental data [file jciinsight-7-141344-s018.pdf]

Supplementary Figure 1

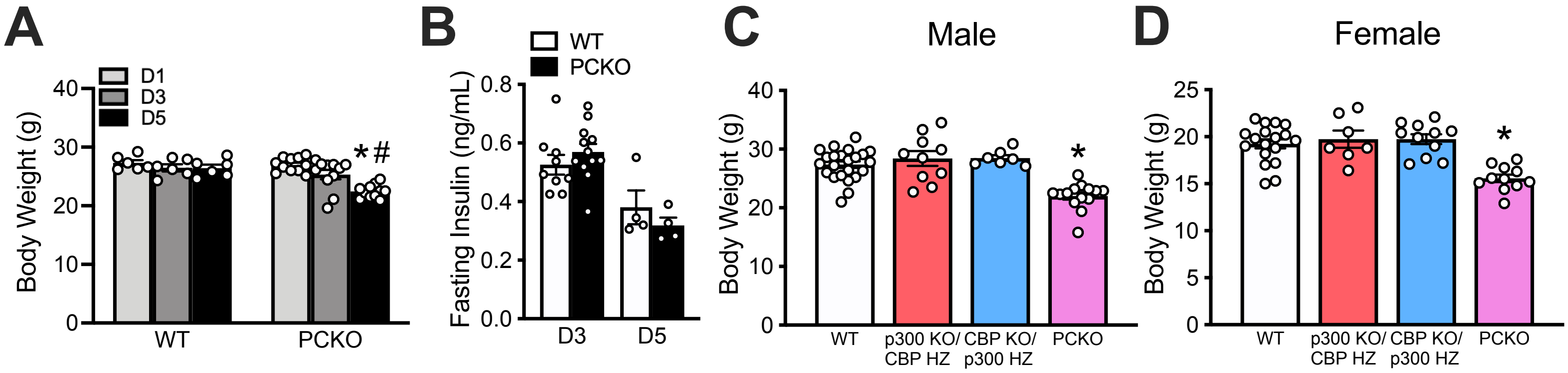

Supplementary Figure 1: PCKO mice have reduced body weight and unchanged fasting insulin. A) Body weights for PCKO and WT mice on one (D1), three (D3), and five (D5) days after starting tamoxifen. *, p<0.05 2-way ANOVA with Sidak's multiple comparison vs WT within the same day and #, p<0.05 2-way ANOVA with Sidak's multiple comparison vs D1 within genotype. WT/PCKO, n = 6/8. B) Fasting insulin concentration for PCKO and WT mice on D3 and D5 after starting tamoxifen, n=4/4. Body weights for C) male and D) female WT, CZ, PZ, and PCKO mice on day five after starting tamoxifen. 1-way ANOVA with Sidak's multiple comparison vs WT. Males: WT;P300 KO/CBP HZ;CBP KO/P300 HZ;PCKO n = 23/10/7/14. Females: WT;P300 KO/CBP HZ;CBP KO/P300 HZ;PCKO n = 18/7/11/11. Data reported as mean±SEM.

Supplementary Figure 2

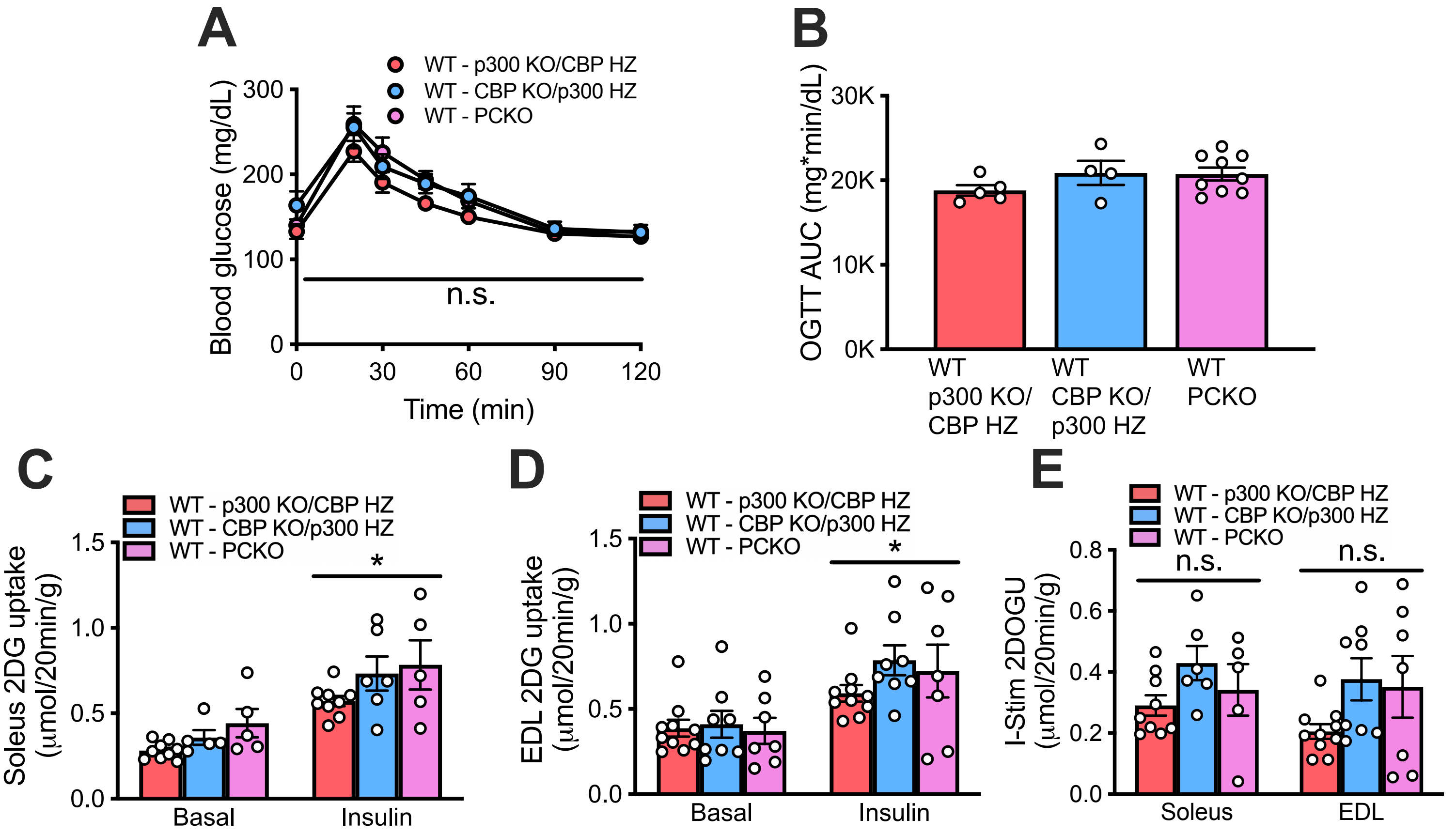

Supplementary Figure 2: Respective WT mice from p300 KO/CBP HZ, CBP KO/p300 HZ, and PCKO lines are not phenotypically different. Male WT mice from respective p300 KO/CBP HZ, CBP KO/p300 HZ, and PCKO mice were assessed at five days after initiating tamoxifen. A) Blood glucose concentrations and B) area under the curve for male WT - p300 KO/CBP HZ, WT - CBP KO/p300 HZ, and WT - PCKO mice during an oral glucose tolerance test (OGTT; 2 g/kg); for p300 KO/CBP HZ;CBP KO/p300 HZ;PCKO n = 5/4/9. 2-way ANOVA, multiple comparison within a time point. Basal 2-deoxy-glucose uptake (2DOGU), and Insulin (0.36 nmol/L) 2DOGU, in isolated C) soleus and D) extensor digitorum longus (EDL) muscles, and E) insulin-stimulated 2DOGU (I-Stim.; calculated as insulin 2DOGU – basal 2DOGU) in isolated soleus and EDL muscles from male WT-p300 KO/CBP HZ, WT - CBP KO/p300 HZ, and WT-PCKO mice; p300 KO/CBP HZ;CBP KO/p300 HZ;PCKO n = 9/6/5. *, $p<0.05$ 2-way ANOVA with Sidak's multiple comparison vs basal within genotype. Data reported as mean±SEM.

Supplementary Figure 3

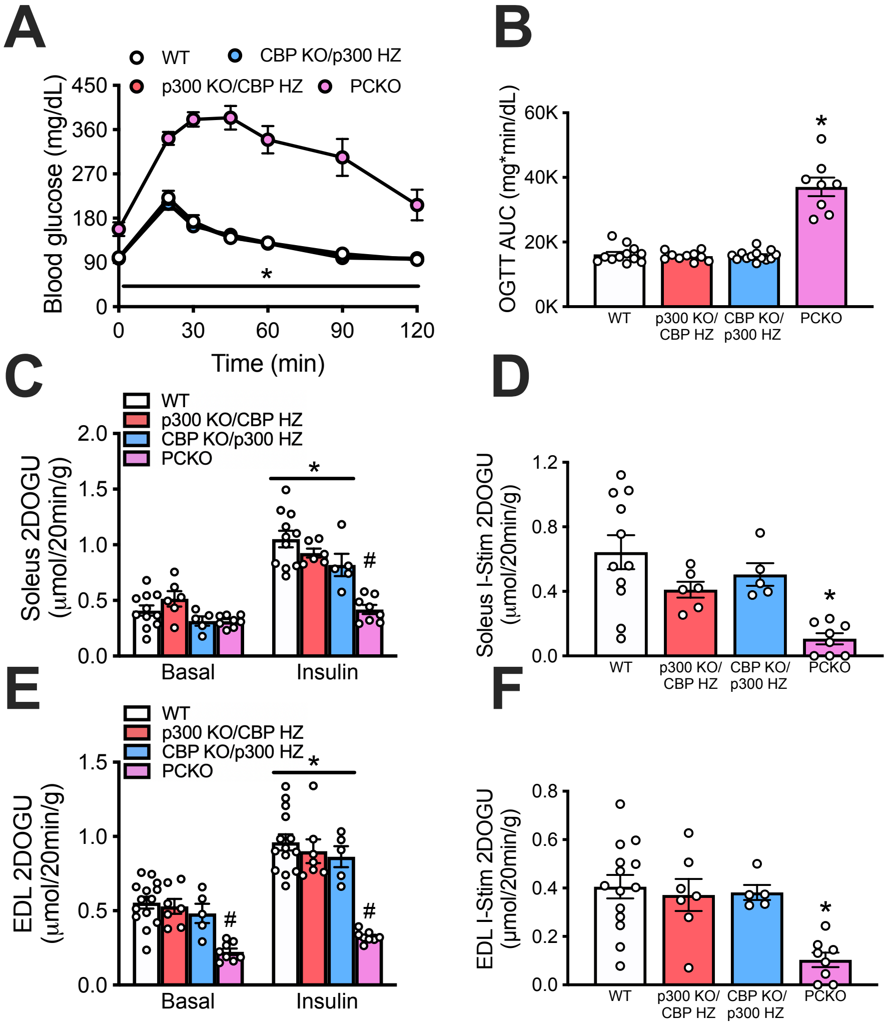

Supplementary Figure 3: PCKO female mice are glucose intolerant and insulin resistant. Female WT, p300 KO/CBP HZ, CBP KO/p300 HZ, and PCKO mice were assessed at five days after initiating tamoxifen. A) Blood glucose concentrations and B) area under the curve (AUC) for female WT, p300 KO/CBP HZ, CBP KO/p300 HZ, and PCKO mice during an oral glucose tolerance test (OGTT; 2 g/kg); for WT;p300 KO/CBP HZ;CBP KO/p300 HZ;PCKO n = 12/10/13/8. *, p<0.05 2-way ANOVA, PCKO vs WT within a time point for OGTT, and *, p<0.05 1-way ANOVA, vs WT for AUC. C-F) Basal 2-deoxy-glucose uptake (2DOGU), Insulin (0.36 nmol/L) 2DOGU, and insulin-stimulated 2DOGU (I-Stim.; calculated as insulin 2DOGU – basal 2DOGU) in isolated C-D) soleus and E-F) extensor digitorum longus (EDL) muscles from female WT, p300 KO/CBP HZ, CBP KO/p300 HZ, and PCKO mice; WT;p300 KO/CBP HZ;CBP KO/p300 HZ;PCKO n = 14/7/5/8. *, p<0.05 2-way ANOVA with Sidak's multiple comparison vs basal within genotype and #, p<0.05 vs WT within basal or insulin. *, p<0.05 1-way ANOVA with Tukey's multiple comparison vs WT for I-Stim. For all data, there were no significant differences between WT mice for the respective lines (p300 KO/CBP HZ, CBP KO/p300 HZ, and PCKO) therefore WT data was collapsed. Data reported as mean±SEM.

Supplementary Figure 4

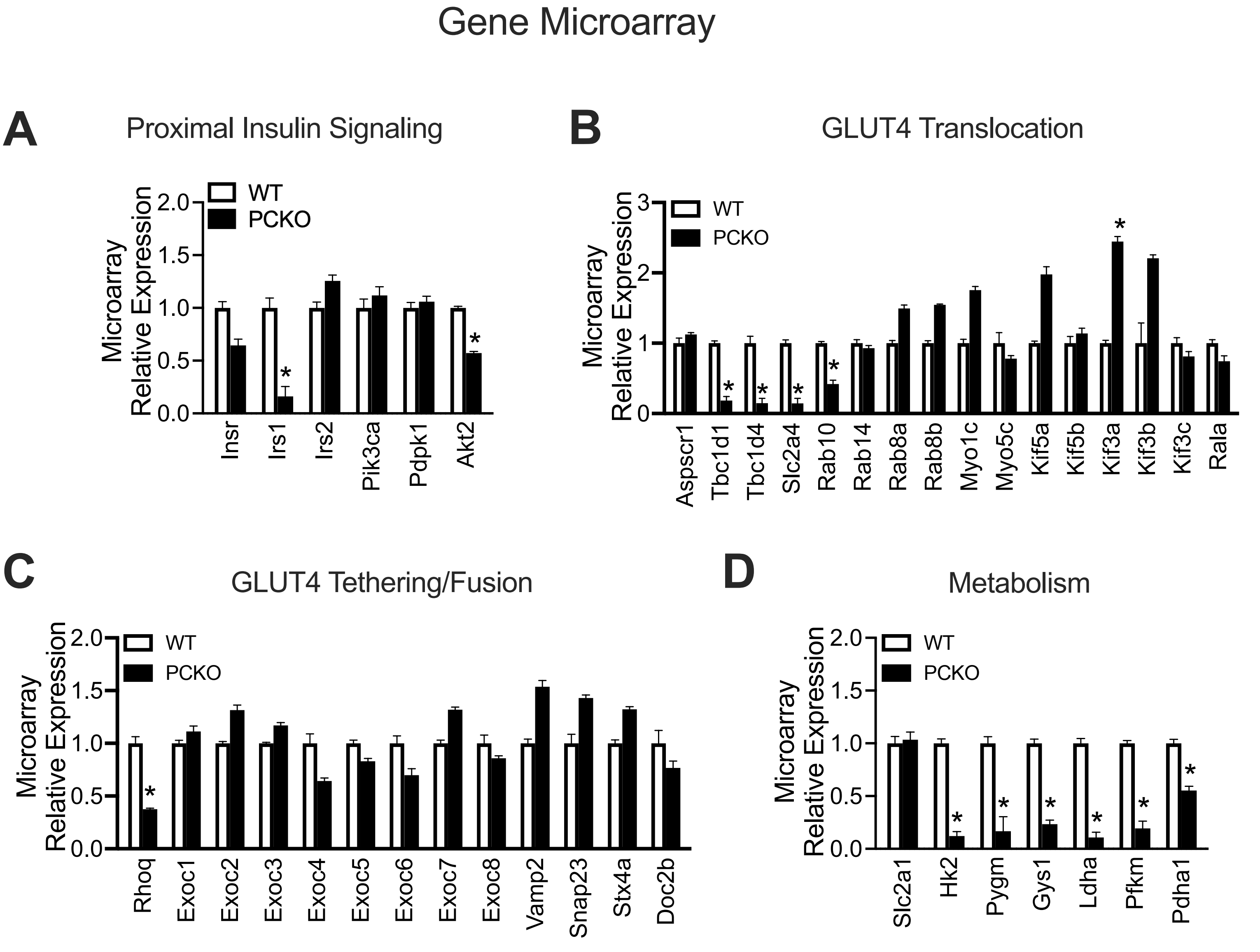

Supplementary Figure 4: Insulin signaling, GLUT4 exocytic translocation, and glucose metabolism mRNA expression in PCKO mice. mRNA expression of genes related to A) insulin signaling, B) translocation of GLUT4, C) tethering/fusion of GLUT4 to the plasma membrane, and D) metabolism in PCKO and WT extensor digitorum longus (EDL) muscles; WT/PCKO n = 4/4. *, FDR <0.1 vs WT. Values are presented relative to WT basal and are expressed as mean ± SEM. Genes are the same ones depicted in Figure 3D.

Supplementary Figure 5

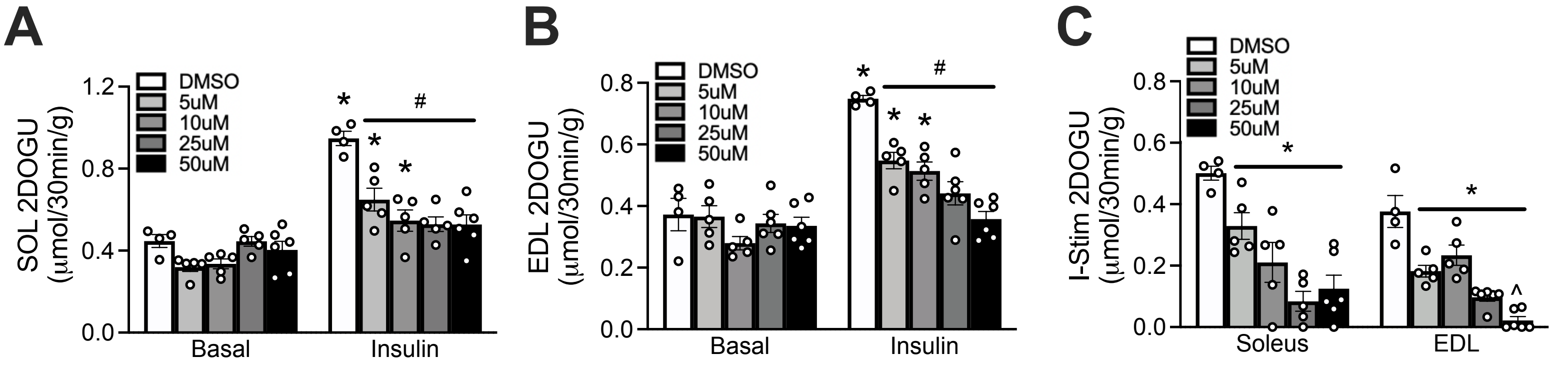

Supplementary Figure 5: Dose response curve for insulin-stimulated glucose uptake in skeletal muscle with C646 treatment. A-B) Basal 2-deoxy-glucose uptake (2DOGU), Insulin (0.36 nmol/L) 2DOGU, and C) insulin-stimulated 2DOGU (I-Stim.; calculated as insulin 2DOGU – basal 2DOGU) in isolated soleus and extensor digitorum longus (EDL) muscles from WT mice pretreated with various concentrations of C646 or DMSO for 1 hour. *, $p<0.05$ 2-way ANOVA with Sidak's multiple comparison vs basal within treatment and #, $p<0.05$ vs WT within basal or insulin. For I-Stim, *, $p<0.05$ 1-way ANOVA main effect and ^, $p>0.05$ one sample *t*-test vs "0". DMSO/5μM/10μM/25μM/50μM n = 4/5/5/5/6. Data reported as mean±SEM.

Supplementary Figure 6

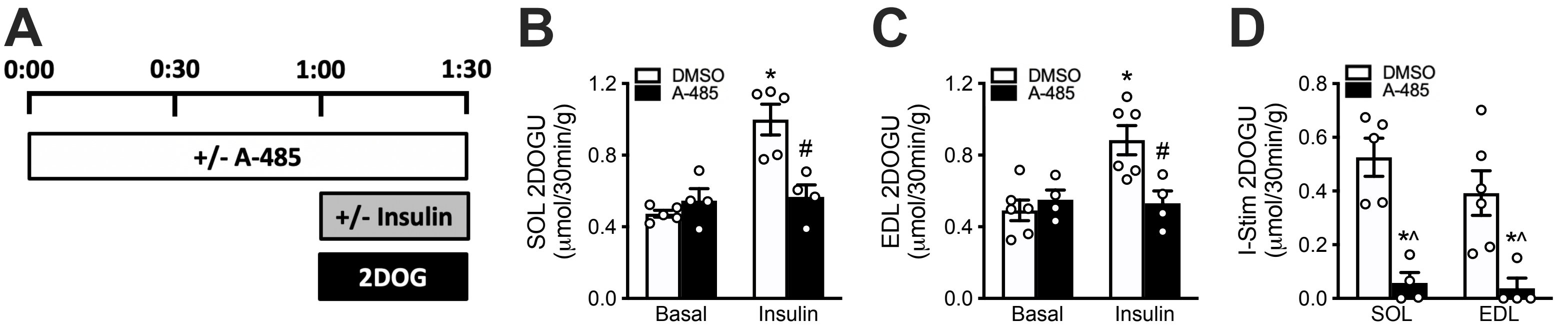

Supplementary Figure 6: Acute inhibition of p300/CBP activity in skeletal muscle with A-485 blocks insulin-stimulated glucose uptake. A) Schematic for experiment. B-C) Basal 2-deoxy-glucose uptake (2DOGU), Insulin (0.36 nmol/L) 2DOGU, and D) insulin-stimulated 2DOGU (I-Stim.; calculated as insulin 2DOGU – basal 2DOGU) in isolated soleus and extensor digitorum longus (EDL) muscles from WT female mice pretreated with either DMSO or 50 μM A-485 for 1 hour. *, $p<0.05$ 2-way ANOVA with Sidak's multiple comparison vs basal within treatment and #, $p<0.05$ vs DMSO within basal or insulin. For I-Stim, *, $p<0.05$ *t*-test and ^, $p>0.05$ one sample *t*-test vs "0". DMSO/ A-485 n= 5/4.

Supplementary Figure 7

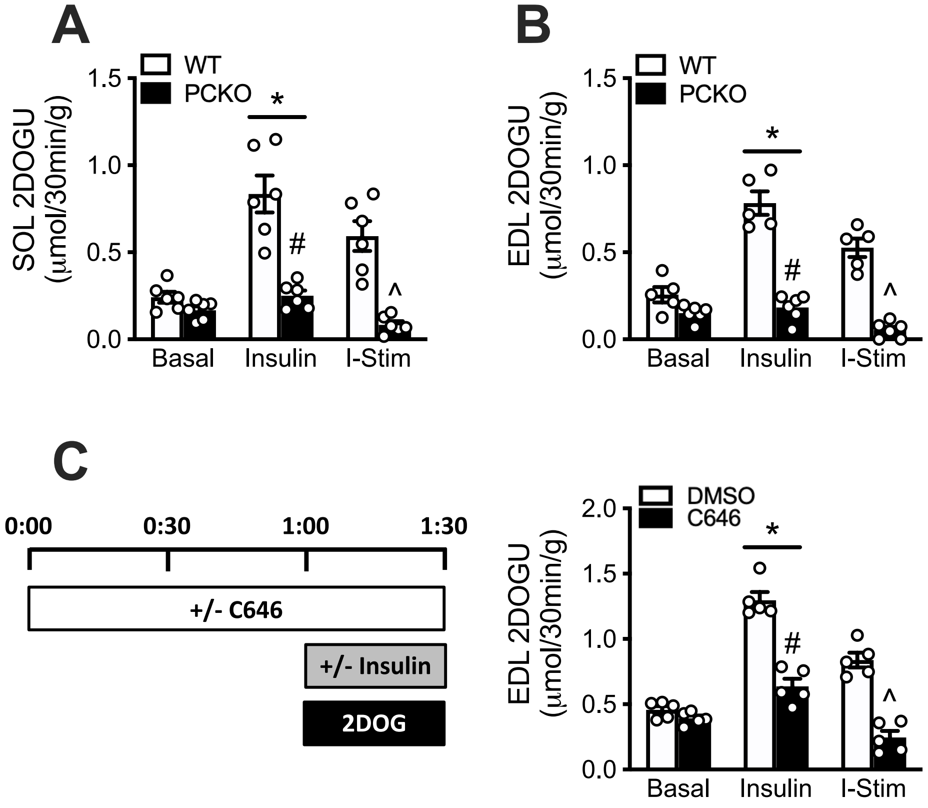

Supplementary Figure 7: Knockout or acute inhibition of p300/CBP in skeletal muscle blocks supraphysiological insulin-stimulated glucose uptake. Basal 2-deoxy-glucose uptake (2DOGU), Insulin (supraphysiological; 6 nmol/L) 2DOGU, and insulin-stimulated 2DOGU (I-Stim.; calculated as insulin 2DOGU – basal 2DOGU) in isolated soleus or extensor digitorum longus (EDL) muscles from A-B) WT and PCKO female mice after 5 consecutive days of tamoxifen, and C) WT female mice pretreated with either DMSO or 50 μM C646 for 1 hour. *, $p<0.05$ 2-way ANOVA with Sidak's multiple comparison vs basal within treatment and #, $p<0.05$ vs DMSO or WT within basal or insulin. For I-Stim, ^, $p<0.05$ *t*-test. WT/PCKO n = 6/6. DMSO/C646 n= 5/5.

Supplementary Figure 8

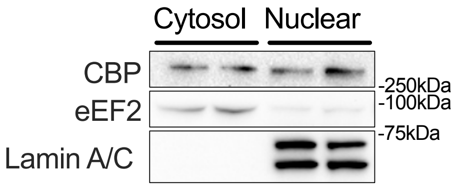

Supplementary Figure 8: CBP is found in the cytosol in skeletal muscle. Subcellular fractionation of WT mouse gastrocnemius muscle with western blot for CBP, eEF2, and Lamin A/C in nuclear and cytosolic fractions, n=2.

Supplementary Figure 9

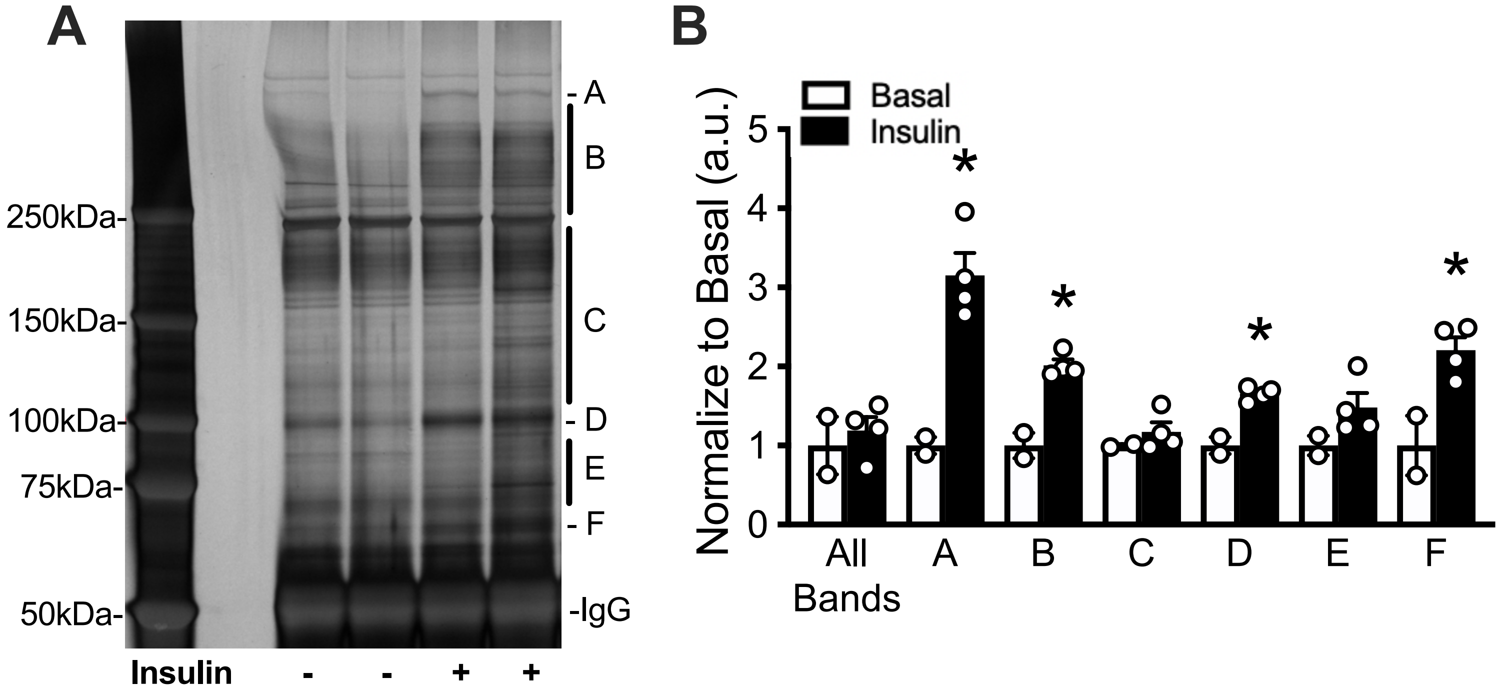

Supplementary Figure 9: Insulin stimulation increases acetylation of proteins in skeletal muscle. A) silver stain and B) quantification of acetyl-lysine (Ac-Lys) immunoprecipitates from basal or insulin stimulated gastrocnemius muscles. Particular bands and regions are quantified as diagramed. *, $p<0.05$ *t*-test. basal/insulin n= 2/4

Supplementary Figure 10

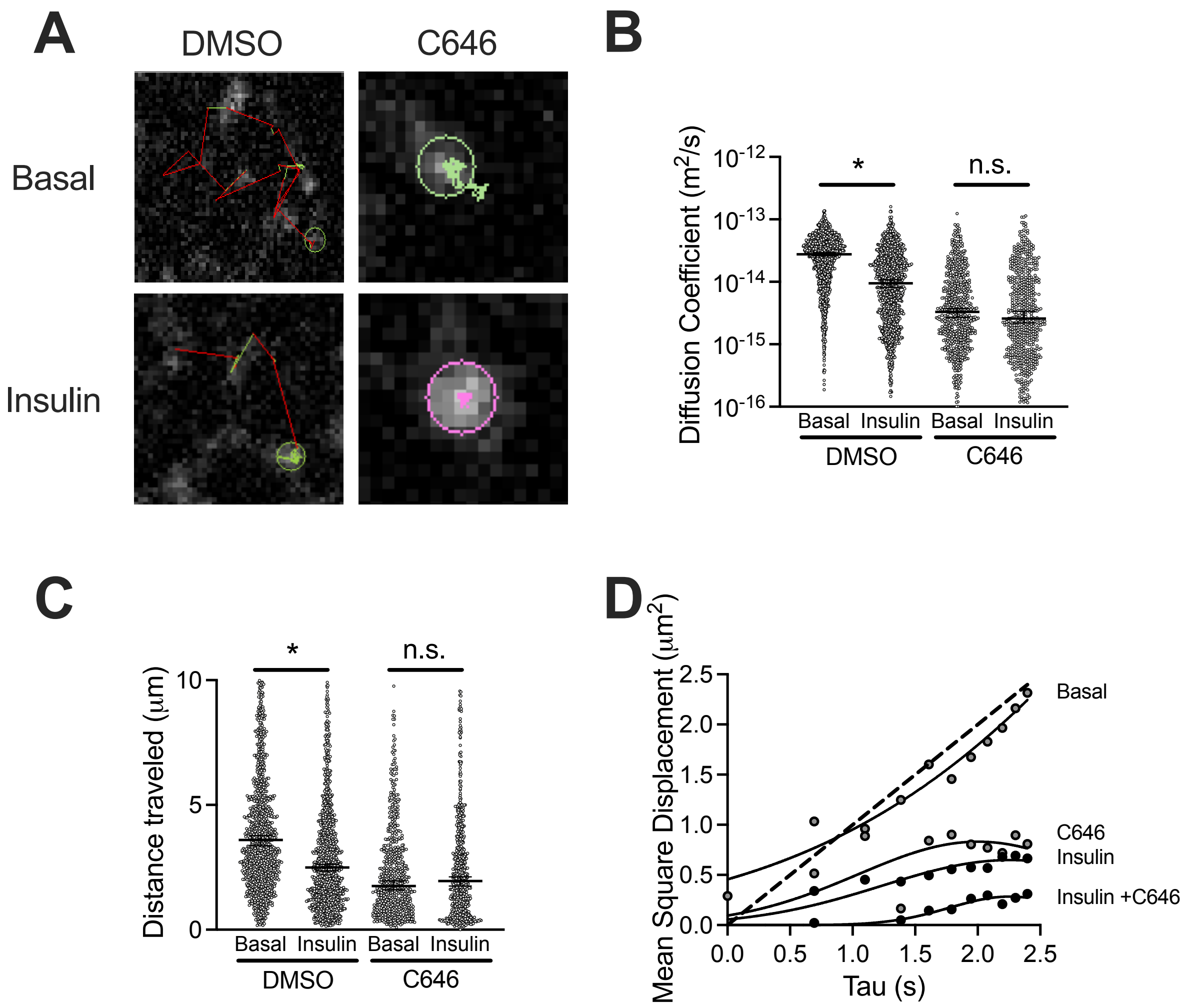

Supplementary Figure 10: Inhibition of p300/CBP activity prevents insulin-stimulated slowing of GLUT4 mobility. A) Single molecule tracking of TIRF imaging using a 60X oil objective (n.a. 1.45) in order to determine B) diffusion coefficient and C) distance traveled of individual GLUT4-GFP with or without insulin or C646 treatment. One representative experiment is shown with 600-1200 puncta per condition. *, $p<0.05$ *t*-test vs basal within condition. Data reported as mean SEM. D) Mean square displacement curves for a representative GLUT4 molecule from each condition. Dashed line represents condition where GLUT4 molecule is moving freely by diffusion (Lizunov et al., 2005, 2013).

Full Blots

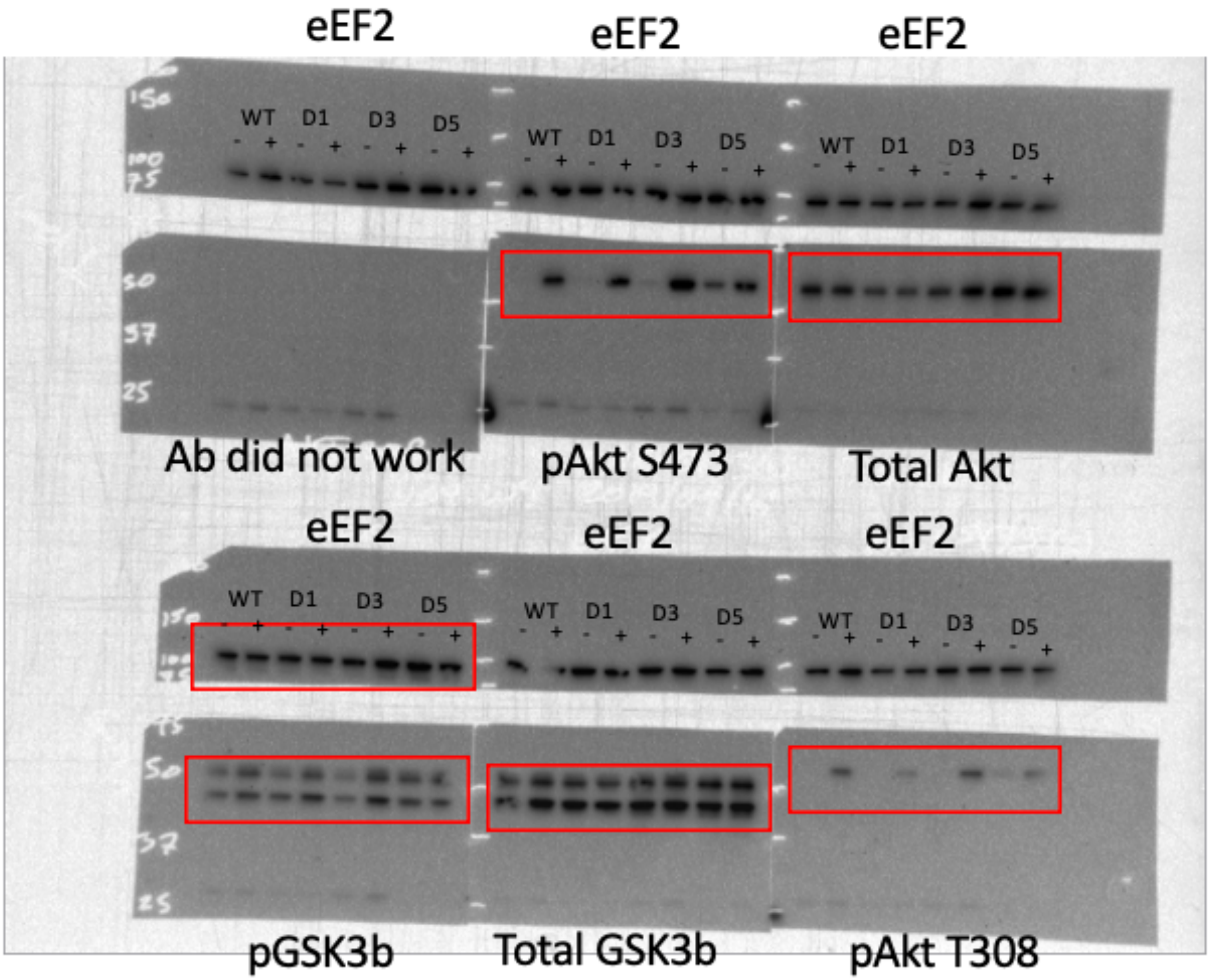

Full unedited gel for Figure 1J: - and + refer to basal and insulin, respectively.

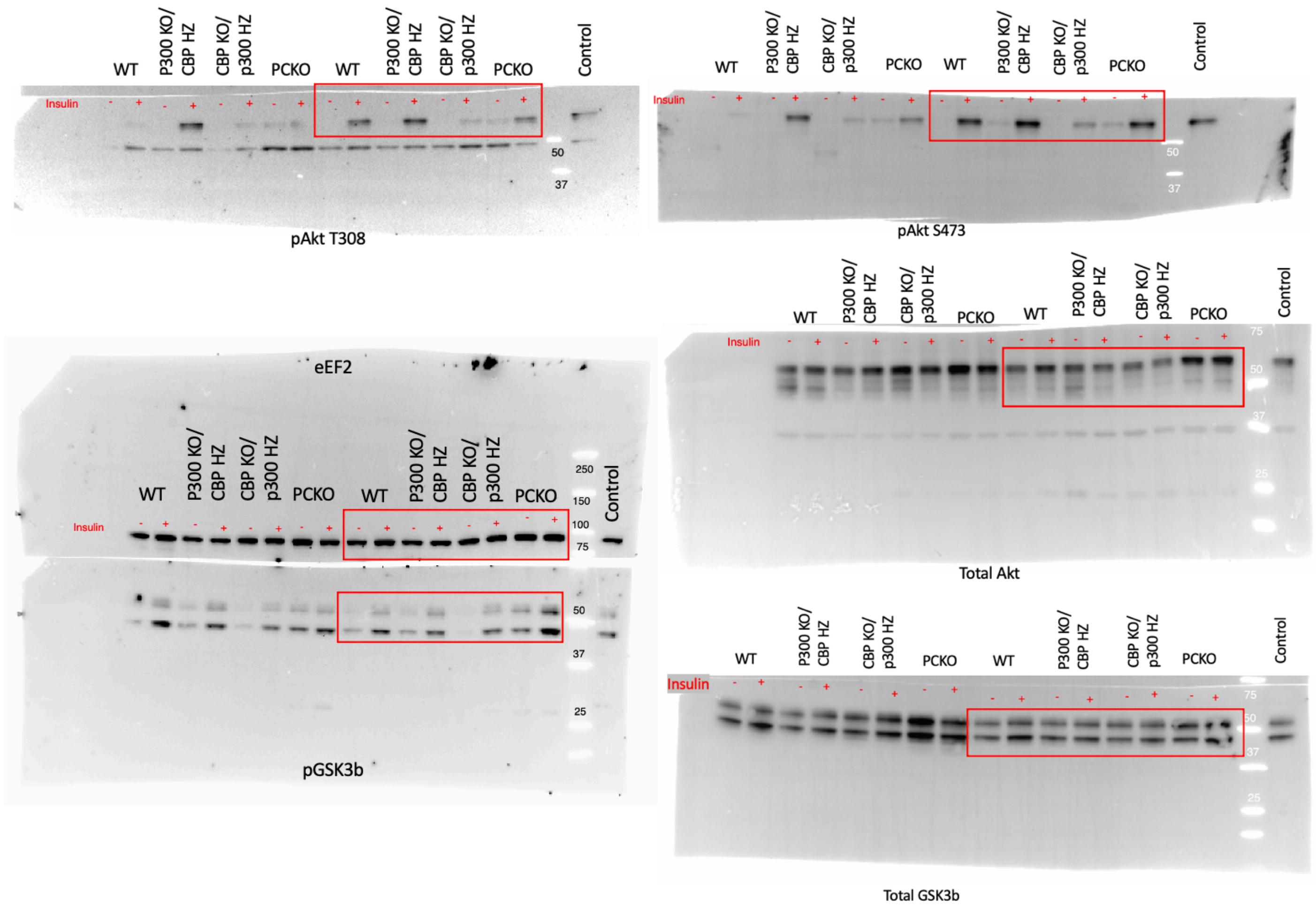

Full unedited gel for Figure 2F: - and + refer to basal and insulin, respectively.

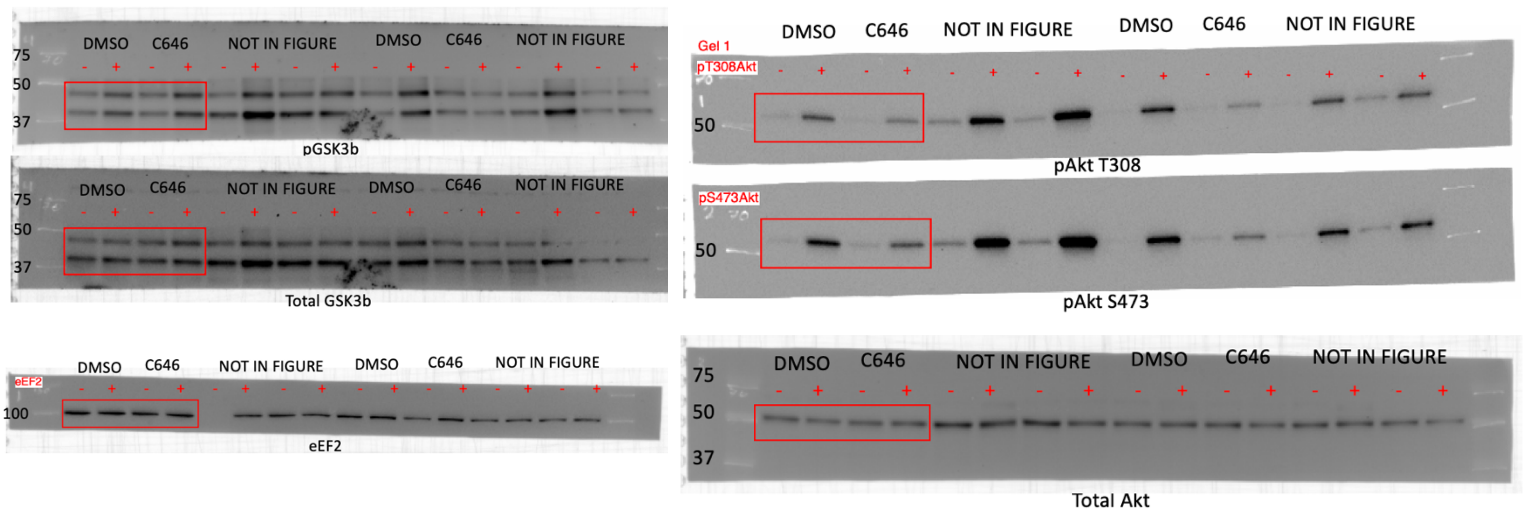

Full unedited gel for Figure 4M: - and + refer to basal and insulin, respectively.

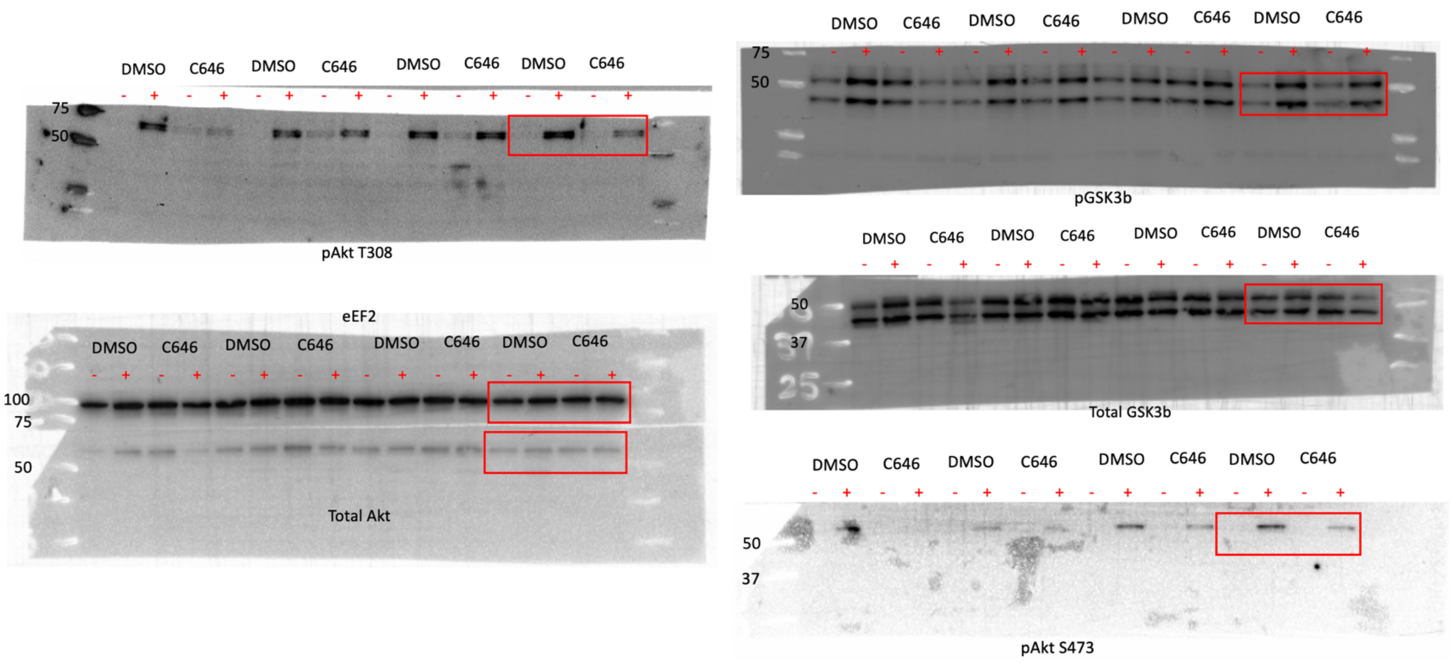

Full unedited gel for Figure 4N: - and + refer to basal and insulin, respectively.

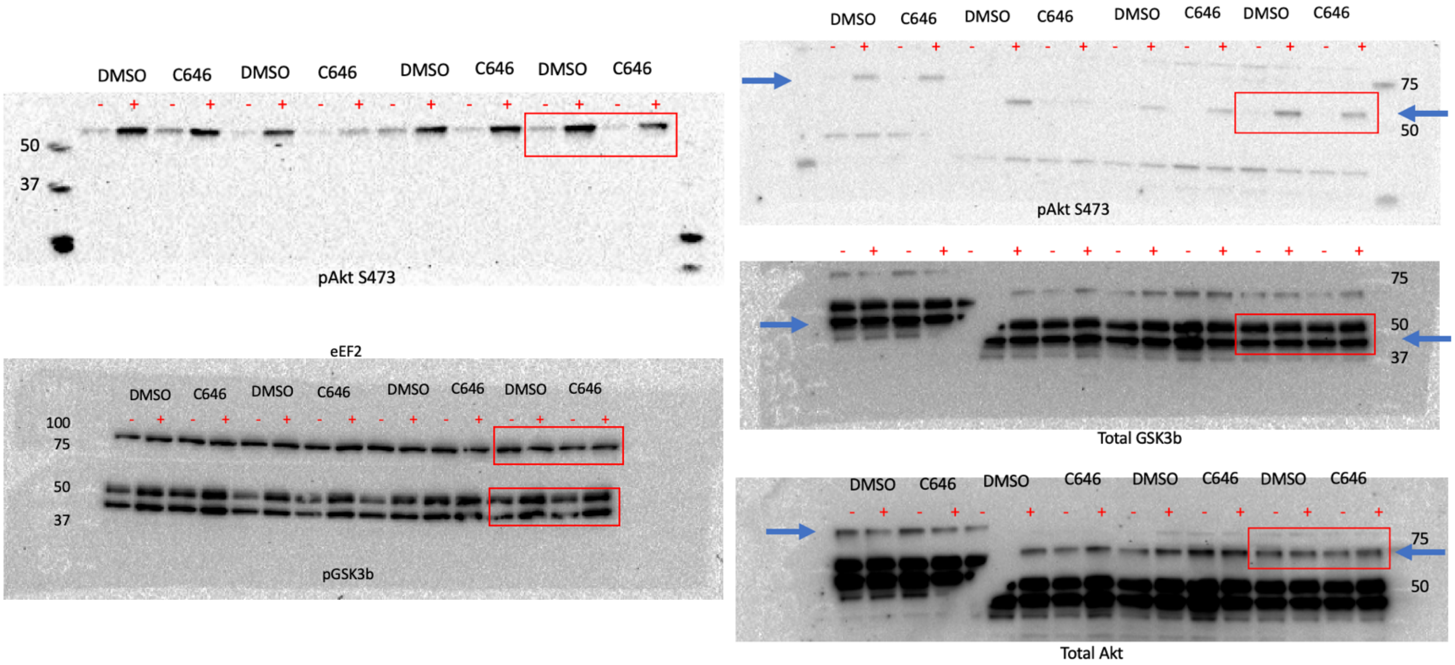

Full unedited gel for Figure 4O: - and + refer to basal and insulin, respectively.

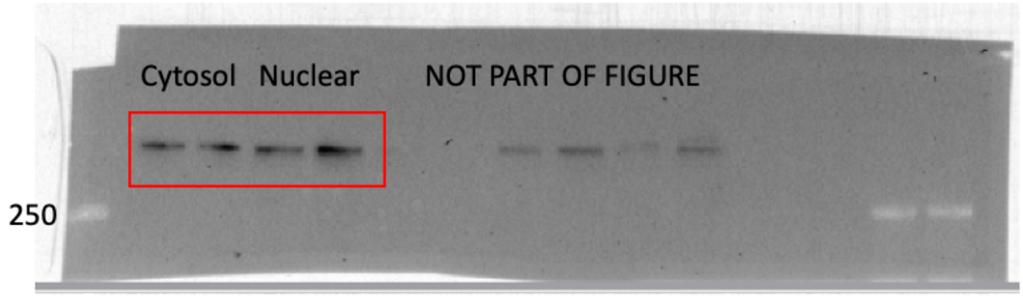

CBP

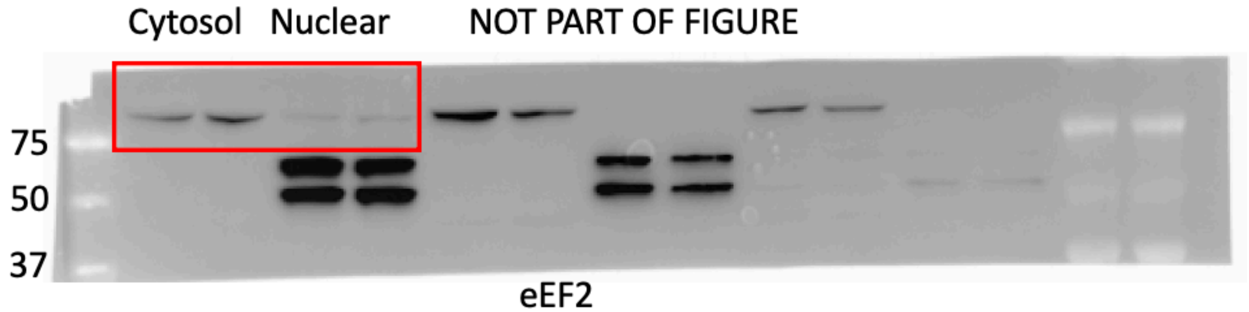

eEF2

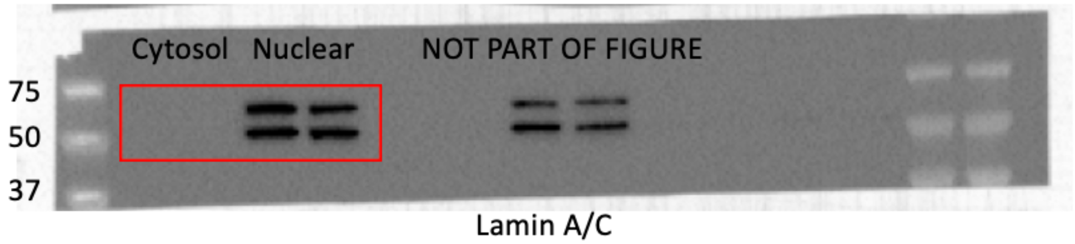

Lamin A/C

Full unedited gel for Supplementary Figure 7

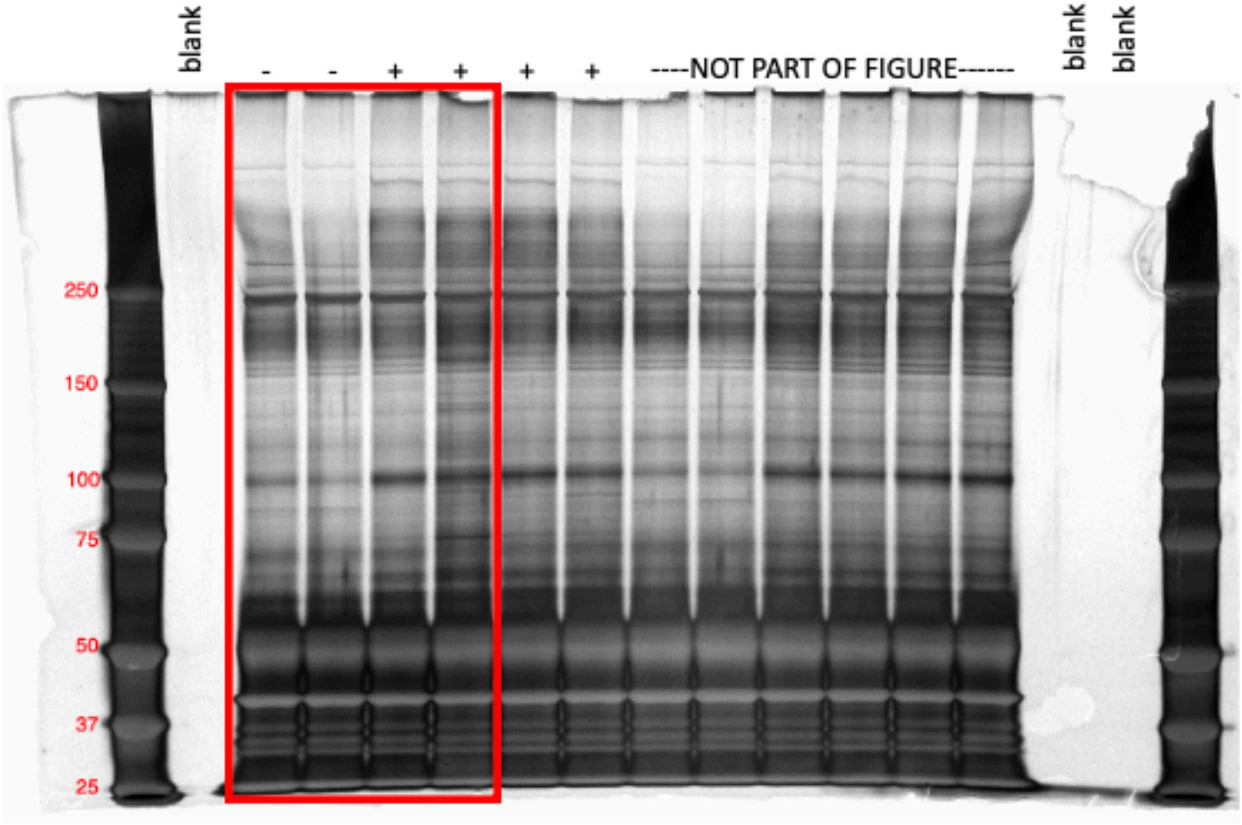

Full unedited gel for Supplementary Figure 8: - and + refer to basal and insulin, respectively.
